# HDAC genes play distinct and redundant roles in *Cryptococcus neoformans* virulence

**DOI:** 10.1038/s41598-018-21965-y

**Published:** 2018-03-26

**Authors:** Fabiana Brandão, Shannon K. Esher, Kyla S. Ost, Kaila Pianalto, Connie B. Nichols, Larissa Fernandes, Anamélia L. Bocca, Marcio José Poças-Fonseca, J. Andrew Alspaugh

**Affiliations:** 1 Department of Cell Biology, Institute of Biological Sciences, University of Brasília, Brasília, Brazil; 2 Department of Medicine/Department of Molecular Genetics and Microbiology, Duke University School of Medicine, Durham, NC USA

## Abstract

The human fungal pathogen *Cryptococcus neoformans* undergoes many phenotypic changes to promote its survival in specific ecological niches and inside the host. To explore the role of chromatin remodeling on the expression of virulence-related traits, we identified and deleted seven genes encoding predicted class I/II histone deacetylases (HDACs) in the *C*. *neoformans* genome. These studies demonstrated that individual HDACs control non-identical but overlapping cellular processes associated with virulence, including thermotolerance, capsule formation, melanin synthesis, protease activity and cell wall integrity. We also determined the HDAC genes necessary for *C*. *neoformans* survival during *in vitro* macrophage infection and in animal models of cryptococcosis. Our results identified the *HDA1* HDAC gene as a central mediator controlling several cellular processes, including mating and virulence. Finally, a global gene expression profile comparing the *hda1Δ* mutant versus wild-type revealed altered transcription of specific genes associated with the most prominent virulence attributes in this fungal pathogen. This study directly correlates the effects of Class I/II HDAC-mediated chromatin remodeling on the marked phenotypic plasticity and virulence potential of this microorganism. Furthermore, our results provide insights into regulatory mechanisms involved in virulence gene expression that are likely shared with other microbial pathogens.

## Introduction

Pathogenic microorganisms must maintain the ability to adapt to environmental changes as well as to the specific cell stresses encountered during interaction with host cells. Chromatin remodeling is one mechanism by which eukaryotic microbes might direct these adaptations in a rapid manner. In eukaryotic cells, genomic DNA is folded with histone and non-histone proteins into chromatin, a highly dynamic organizational structure. The basic subunit of chromatin is the nucleosome, consisting of DNA surrounding two H3/H4 histone protein heterodimers and two H2A/H2B histone heterodimers^1,2^. These histone proteins are the targets of different post-translational modifications, leading to changes in the chromatin structure. The degree of chromatin remodeling resulting from these histone modifications regulates gene expression, offering a more rapid mechanism of adaptation than spontaneous genetic mutations^3–5^.

One of the best studied post-translational histone modification is the variable acetylation that occurs at lysine residues. Histone acetylation and deacetylation are regulated by the activities of opposing enzymes: the histone acetyltransferases (HATs) and histone deacetylases (HDACs)^6^. Chromatin remodeling is involved in several cell processes such as stress response, adaptation, immune response and carcinogenesis^7–12^. However, chromatin remodeling mechanisms are poorly understood in pathogenic microorganisms, particularly in the context of virulence.

HDACs catalyze the removal of acetyl groups, leading to chromatin condensation^13,14^. Therefore, these enzymes play crucial roles in regulating gene expression as they modulate the accessibility of chromatin to transcriptional regulators and other regulating factors^14^. HDACs are evolutionarily conserved and are found in plants, fungi, and animals, as well as archaea and eubacteria^15,16^. They have been associated with epigenetic phenotypic changes in many fungal species such as *Ustilago maydis*^17^, *Aspergillus nidulans*^18^, *Candida albicans*^19–21^, *Schizosaccharomyces pombe*^22,23^, *Saccharomyces cerevisiae*^24^, and *Cryptococcus neoformans*^25,26^. It has also been reported that HDACs play important roles in virulence-related processes and morphological changes in some fungi. For example, the plant fungal pathogen *U*. *maydis* displays altered development and pathogenesis in the setting of altered HDAC activity^17^. Additionally, the human fungal pathogen *C*. *albicans* requires intact HDAC function for the yeast-hyphal transition that is central to its pathogenesis^20^.

Phylogenetically, fungal HDACs are divided into three main classes. The “classical” HDAC family proteins fall into two classes: class I (Hos2- and Rpd3-like proteins) and class II (Hda1-like proteins)^15,27,28^. The “non-classical” or Class III HDACs are Sir2-like proteins^16^. The central enzymatic domains of Class I and II HDACs are similar to one another, containing a central Zn^2+^ atom^29^. The class III enzymes are more divergent and are nicotinamide adenine dinucleotide (NAD)-dependent^30^. The number of HDACs of each class encoded in the genome can vary widely between species. For example, the model fungi *S*. *cerevisiae* and *S*. *pombe* contain differing numbers of HDAC genes. The budding yeast *S*. *cerevisiae* has three class I HDACs (Rpd3, Hos2 and Hos1) and two class II HDACs (Hda1 and Hos3). In contrast, in the fission yeast *S*. *pombe* there are two class I HDACs (Clr6 and Hos2) and one class II HDAC (Hda1)^6,27^.

In previous experiments, we tested the effects of chemical inhibitors of Class I and II HDACs (Sodium butyrate and Trichostatin A, respectively) on the expression of virulence-associated phenotypes in the human fungal pathogen *C*. *neoformans*. This encapsulated yeast is frequently found in the environment in association with decaying vegetation^31^. However, when encountered by people with compromised immune systems, especially persons with AIDS^32^, this microorganism can cause a lethal infection of the central nervous system^33^. In addition to its clinical importance, this fungal pathogen displays a remarkable degree of phenotypic plasticity in response to host and environmental cues (reviewed in^34^). We demonstrated that these HDAC inhibitors affected the expression of several microbial phenotypes, including growth rate at 37 °C, expression of the anti-phagocytic polysaccharide capsule, and production of the antioxidant melanin pigment^35^.

Here, we conducted a complementary genetic investigation of HDACs in *C*. *neoformans*. Using available genome data, we performed a comprehensive characterization of Class I and II HDAC homologues in this species. By creating targeted mutations of each of these HDAC genes, we tested our hypothesis that individual HDAC proteins would control specific aspects of *C*. *neoformans* adaptation to various environmental cues.

## Results

### Identification and characterization of *C*. *neoformans* Class I and II HDAC genes

**Figure 1:**
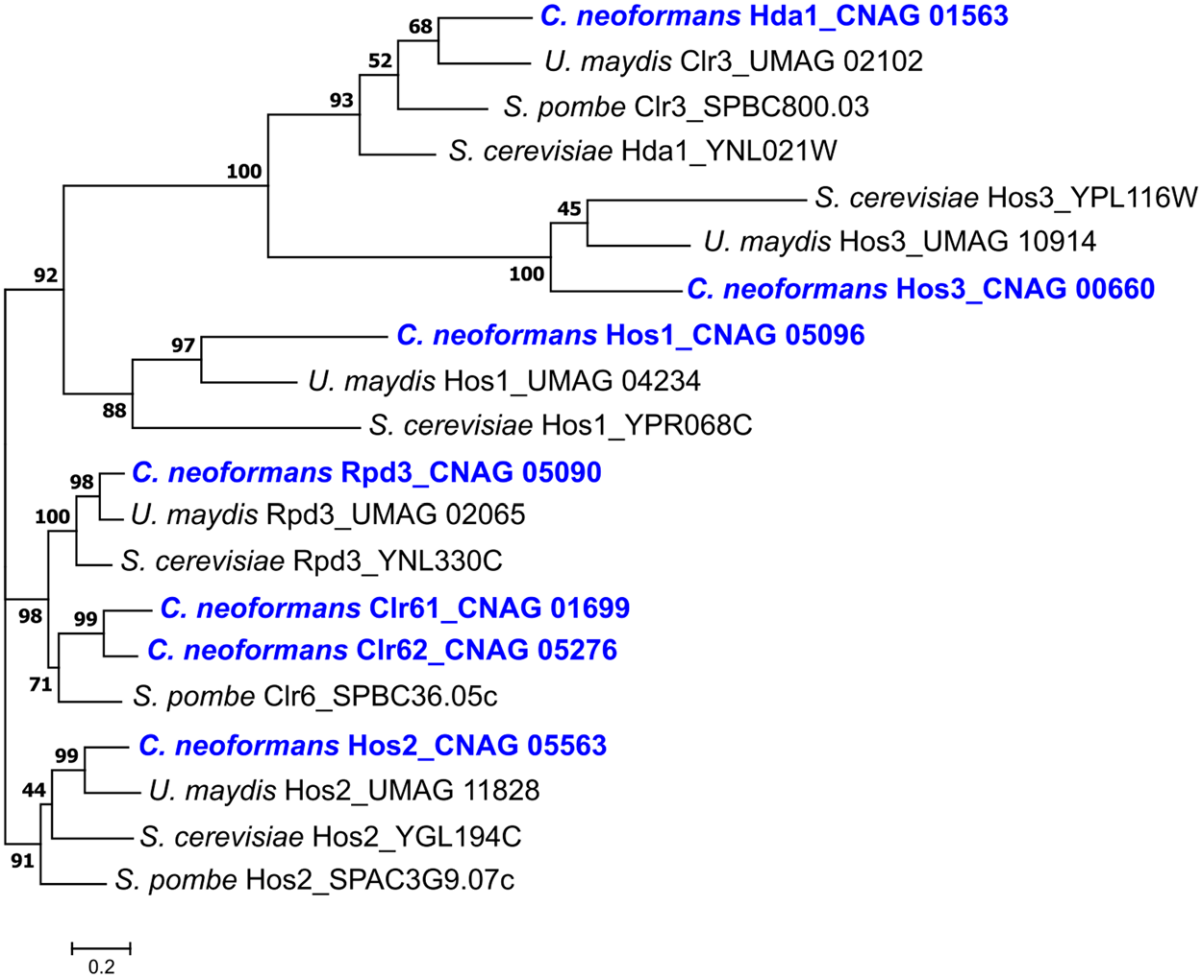
Different fungal species contain varying numbers of genes encoding Class I and Class II histone deacetylases (HDACs). Maximum likelihood statistical method was used to demonstrate phylogenetic relationships for the predicted protein sequences for Class I and Class II HDACs in *Cryptococcus neoformans*, *Saccharomyces cerevisiae*, *Schizosaccharomyces pombe* and *Ustilago maydis*. The phylogenetic tree was created in MEGA7 with a WAG + G + I model and gamma shape parameters with bootstrap test of phylogeny applied with 500 replicates. Scale bar: 0.2 amino acid substitutions per site.

We used known HDAC gene sequences from three divergent fungal species (*S*. *cerevisiae*, *S*. *pombe*, and *U*. *maydis)* to conduct a genome-wide BLAST search and phylogenetic analysis of putative Class I and II HDAC genes in *C*. *neoformans*. Seven such genes were identified in the genome of the clinical *C*. *neoformans* strain H99 (Fig. 1**)**. Two of the seven *C*. *neoformans* HDAC genes, encoding predicted Class I enzymes, had already been identified: *HOS2* (CNAG_05563) and *RPD3* (CNAG_05690)^25^. The other five predicted HDAC genes were named according to their closest relative in *S*. *cerevisiae* or *S*. *pombe*: Class I: *HOS1* (CNAG_05096), *CLR61* (*CLR6* homologue-1; CNAG_01699), and *CLR62* (*CLR6* homologue-2; CNAG_05276); Class II: *HDA1/CLR3* (CNAG_01563), and *HOS3* (CNAG_00660). The *C*. *neoformans HDA1/CLR3* gene was discussed in a recent manuscript as *CLR3*, encoding a possible histone modifier responsible for coordinating with Polycomb proteins to assist in the repression of subtelomeric gene expression^26^. However, two publications assigned the “*CLR3*” gene name to a distinct *C*. *neoformans* gene encoding a B-zip transcription factor required for capsule formation (CNAG_00871)^36,37^. Therefore, to avoid confusion in nomenclature, we refer to the CNAG_01563 gene by its closest homologue in *S*. *cerevisiae*, *HDA1*. All seven putative *C*. *neoformans* proteins identified in this manner possess domains characteristic of class I or II HDACs.

To ensure that these putative HDAC genes were actually expressed, we compared transcript levels for each gene after incubation of the wild-type strain in rich YPD medium versus incubation in minimal medium. The later condition was chosen since it induces a nutritional stress associated with capsule and melanin biosynthesis, two phenotypes that were impaired by pharmacological HDAC inhibition^35^. All seven HDAC genes were expressed and showed increased transcript accumulation when transitioned to minimal medium (Fig. S1).

To evaluate whether HDACs were relevant for *C*. *neoformans* virulence, we generated deletion mutants for each class I and II HDAC gene in strain H99 *MAT***a** and *MAT****α*** genetic backgrounds. The *HOS2* and *RPD3* genes were previously described to be important for *C*. *neoformans* virulence in a murine model of cryptococcal infection^25^. We re-created these two mutants independently and used them as controls for comparison to the other mutant strains in our experimental conditions.

### HDAC genes are required for stress tolerance and expression of virulence-associated phenotypes

**Figure 2:**
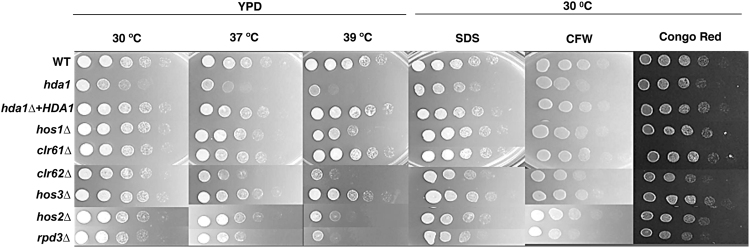
Temperature and cell stress-related phenotypes of the HDAC mutants. 5-fold serial dilutions for the indicated strains were spotted onto various media at the indicated temperatures. Growth was assessed after 48 hours of incubation. SDS (sodium dodecyl sulfate); CFW (calcofluor white).

HDACs regulate different processes involved in adaptation and stress response^17,23^. We therefore tested the HDAC mutants for various phenotypes related to virulence and microbial differentiation (summarized in Table 1). Thermotolerance at 37 °C was compromised for the *hda1Δ*, *hos1Δ*, *clr62Δ*, *hos2Δ* and *rpd3Δ* HDAC mutants, being even more pronounced at 39 °C (Fig. 2). Additionally, growth inhibition in the presence of cell surface stresses (SDS, Calcofluor White and Congo Red) was observed for the *hda1Δ*, *clr62Δ* and *rpd3Δ* mutants, suggesting a defect in cell integrity in these strains (Fig. 2).

**Table 1:** Phenotypic comparisons of HDAC mutants.

Strain	39 °C growth	Cell wall stressors	Capsule	Melanin	Protease activity	Survival in macrophages
Class I HDAC						
*rpd3∆*	−	−	+	+	+	−
*hos2∆*	−	+	+	−	−	−
*hos1∆*	−	+	++	+	+	−
*clr61∆*	+	+	+	+	+	++
*clr62∆*	−	−	−	+	+	−
Class II HDAC						
*hda1∆*	−	−	−	−	−	−
*hos3∆*	+	+	−	+	+	−

Summary of *in vitro* data presented in Results (“−” indicating defective phenotype, “+” indicating a phenotype similar to wild-type, and “++” indicating enhanced compared to wild-type).

**Figure 3:**
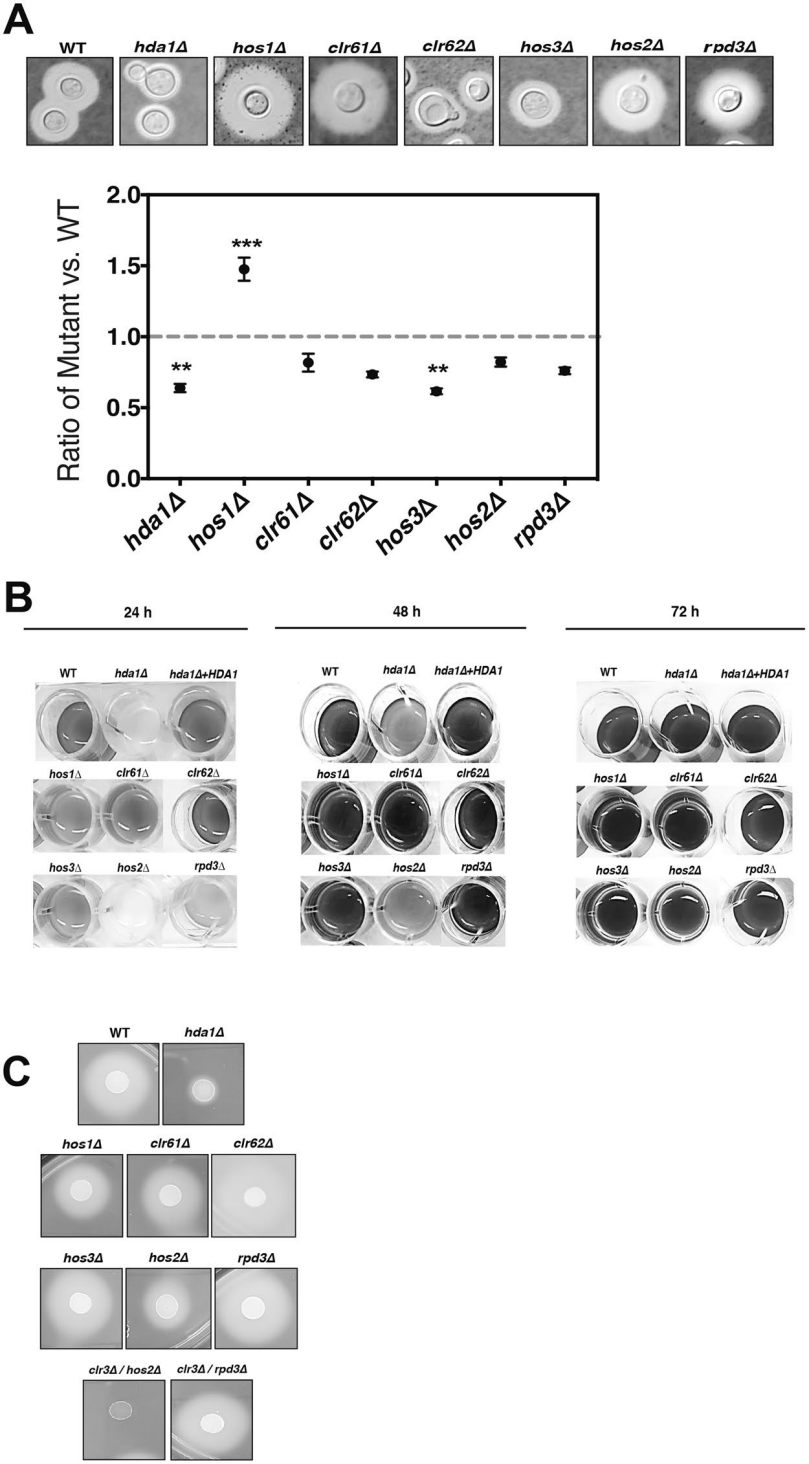
HDACs control *C*. *neoformans* virulence-associated phenotypes. (**A**) Capsule. Each strain was incubated in CO_2_-independent tissue culture medium for 72 hours to induce capsule formation. Capsule size was assessed using India ink counterstaining, and representative cells are displayed Capsule volume was quantified by assessing the packed cell volume of normalized cell suspensions, expressed as a ratio to wild-type. Data points represent averages of triplicate samples (+/− standard error). (**B**) Melanin. Indicated strains were incubated in minimal media with 1 mM L-DOPA at 30 °C, 150 rpm. Melanin production was assessed visually at 24, 48, and 72 hours. (**C**) Extracellular protease production. Indicated strains were spotted onto BSA agar and incubated at 30 °C for 3 days. The presence of a peripheral clear halo indicates protease activity.

Since a marked reduction of *C*. *neoformans* capsule size was observed after chemical HDAC inhibition^35^, we hypothesized that specific HDAC mutants would correspondingly exhibit a hypocapsular phenotype. After incubation in capsule-inducing conditions, the *hda1Δ*, *clr62Δ* and *hos3Δ* mutants showed a significant reduction in capsule expansion (Fig. 3A), an overlapping but non-redundant set of mutants compared to those with stress-induced growth defects (Fig. 2). Capsule changes were assessed both by direct microscopy with India ink counter-stain as well as by quantitative determination of packed cell volume as a surrogate measure of total cell volume (Fig. S2A). In contrast, the *hos1Δ* mutant maintained a larger surface capsule than wild-type, suggesting that HDACs can play different or opposing functions in pathogen-related phenotypes.

Despite its reduction in surface capsule expression, the *hda1Δ* mutant displayed an apparent increase in total cell volume, perhaps a sign of delayed cell cycle progression (Fig. S2B). In this haploid yeast species, flow cytometry of propidium iodide-stained *hda1Δ* mutant cells demonstrated an enrichment in the population of cells with a 2n DNA content after incubation at 37 °C, suggestion a shift in cells in the G2/M stage of the cell cycle (Fig. S2C). Interestingly, we observed a similar delay in cell cycle progression when we treated *C*. *neoformans* cells with the Class I/II HDAC inhibitor sodium butyrate (NaBut)^35^.

Melanin production is an important virulence factor for *C*. *neoformans*, protecting the fungal cells from free radical-induced damage^38^. When incubated in melanin-inducing medium, we observed a marked delay in melanin production for the *hda1Δ* and *hos2Δ* mutants (Fig. 3B). Prolonged incubation resulted in eventual melanization of all HDAC mutant strains. The melanin defect in the *hda1Δ* mutant was fully suppressed in the *hda1Δ* + *HDA1* reconstituted strain (Fig. 3B). These two melanin-deficient strains also produced decreased extracellular proteases, as determined by a reduced halo of clearing on BSA medium (Figs 3C, S3). Secreted proteases likely promote *C*. *neoformans* survival during interaction with host cells.

### *HDA1* plays a role in *C*. *neoformans* sexual reproduction

In other fungal species such as *U*. *maydis* and *S*. *pombe*, deletion of HDAC genes results in defective mating, emphasizing the importance of chromatin remodeling in sexual reproduction and development^6,17,39,40^. Additionally, the functional role of many HDACs has been linked to the yeast-to-hyphae transition in *C*. *albicans*, another form of fungal morphological transition^41,42^. We previously observed reduced *C*. *neoformans* mating hyphae production after chemical HDAC inhibition^35^, indicating that these enzymes are also likely involved in the mating and hyphal developmental processes in *C*. *neoformans*.

**Figure 4:**
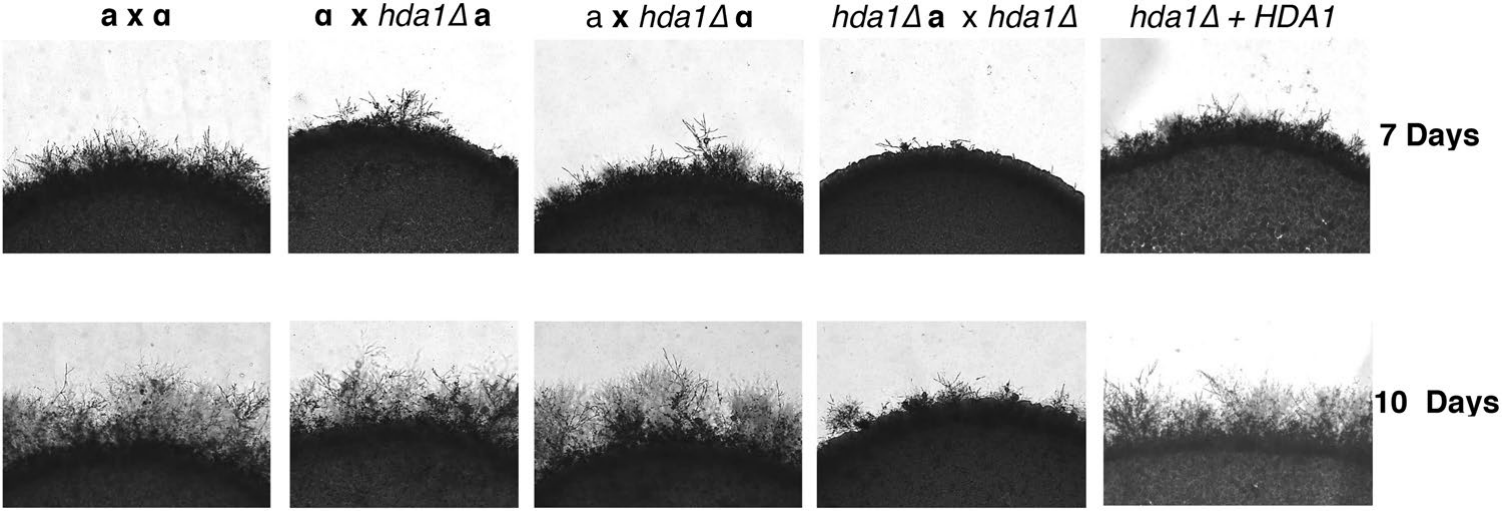
*HDA1* is required for mating. Overnight cultures of wild-type, *hda1Δ* mutant and *hda1∆* + *HDA1* reconstituted strains of opposite mating type (*MAT****a*** or *MATα*) were mixed as indicated in equal amounts onto MS agar and incubated at room temperature, protected from the light. The edges of the mating mixtures were assessed for mating hyphae at 7 and 10 days (100×).

The individual HDAC mutants were crossed with a wild-type mating partner, and mating hyphae formation was monitored daily. For most HDAC mutants, no alterations in mating were observed (Fig. S4). However, the *hda1Δ* mutant showed a noticeable reduction in hyphae formation compared to wild-type (Fig. 4). This reduction in mating hyphae production is subtle when observed in unilateral crosses (*hda1Δ* × wild-type), but it is more pronounced in a bilateral cross (*hda1Δ* **a** × *hda1Δ* **α**) (Fig. 4). The impaired mating phenotype is completely restored to wild-type levels in the *hda1Δ *+* HDA1* reconstituted strain. The *HDA1* homolog also has been implicated in chromatin dynamics at the mating-type locus and in promoting epigenetic stability of heterochromatin in *S*. *pombe*^23^.

### HDACs are required for *C*. *neoformans* pathogenicity

**Figure 5:**
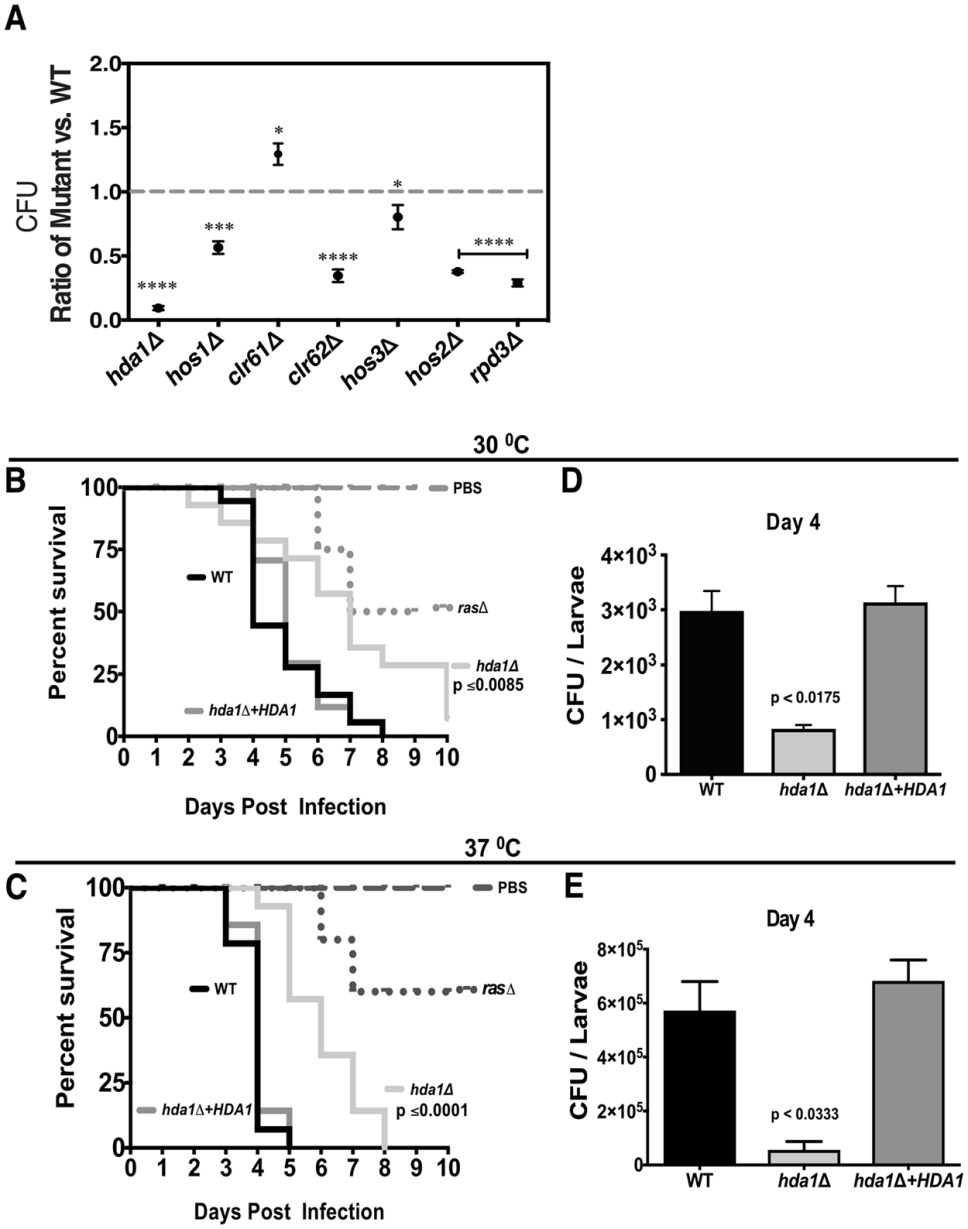
The Hda1 HDAC protein is required for virulence in surrogate models of infection. (**A**) HDACs and survival in macrophages. Cells of each strain were co-incubated with J774A.1 macrophages (MOI: multiplicity of infection of 1:1). Macrophages were pre-activated with PMA (phorbol myristate acetate) and yeasts were opsonized with the 18B7 anti-GXM antibody. Viable colony-forming units (CFU) after 18 hours of co-cultures were assessed by quantitative culture, and the graphs represent the average ratio of each mutant strain normalized to the wild-type control (dashed line). Error bars represent standard errors of the mean. Statistical test: One-way ANOVA with Dunnett’s multiple-comparison test used to compare the means of results from three independent experiments. ****p < 0.0001; ***p < 0.001; *p < 0.05. (**B,C**) Galleria mellonella. The indicated strains were inoculated into larvae of the greater wax moth *G*. *mellonella*, and survival was monitored at 30 °C (B) and 37 ^°^C (C). Mock infections with PBS injections were used as uninfected controls. Strains: Wild-type, *hda1∆* mutant, *hda1∆* + *HDA1* reconstituted strain and the *ras1∆* hypovirulent mutant^43^. Statistical test: Kaplan-Meier method. **(D,E)** In a separate experiment, 5 larvae from each group were assessed for fungal burden by quantitative culture on day 4 post infection. Statistical test: One-way ANOVA with Dunnett’s multiple-comparisons test.

To assess the virulence of the HDAC mutants, we first examined the intracellular survival of all HDAC mutant strains in co-culture experiments with J774.1 murine macrophages. After 18 h of co-incubation, macrophages were lysed and the number of colony-forming units (CFUs) of surviving yeast cells was determined by quantitative culture. Phagocytosis rates did not significantly differ between these strains and wild-type, suggesting that altered intracellular survival most likely accounted for different CFUs after co-culture (data not shown). Although most of the mutants showed some degree of reduced survival in macrophages, the *hda1Δ* mutant was the most attenuated, consistent with its defects in multiple virulence-associated phenotypes. Interestingly, the *clr61Δ* mutant demonstrated better survival compared to wild-type in this *in vitro* model of host-pathogen interaction (Fig. 5A).

We also used a well-characterized invertebrate model of host-fungal interaction to assess the virulence of the HDAC mutants in the larvae of the greater wax moth *Galleria mellonella*. This model assesses survival of the infected insect host as well as measuring persistence of the infecting fungal cells. Additionally, since this infection can be performed at both ambient temperature at 30 °C and 37 °C, we were able to distinguish the effects of altered thermotolerance from other contributors to virulence. The *clr62∆* mutant was fully virulent in the Galleria infection model, even though it displayed reduced survival in macrophage co-culture (Fig. S5A). These observations suggest that the *clr62∆* thermotolerance defect, and not its other more modest *in vitro* mutant phenotypes, explains its altered interaction with macrophages. Consistent with their persistence in macrophages, the *clr61∆* and *hos3∆* mutants displayed no detectable alteration in virulence in the *Galleria* infection model, with larvae succumbing to the infection at a similar rate as those infected with the isogenic wild-type strain (Fig. S5B,C). Moreover, quantitative cultures indicated higher levels of persistence of the *clr61∆* mutant compared to wild-type, similar to its enhanced ability to survive within activated macrophages (Fig. S5F). Also consistent with the macrophage data, the *hos2∆* and *rpd3∆* mutants displayed reduced virulence in *Galleria*, with statistically significant reductions in host death rate due to infection (Fig. S5D,E). The *hda1∆* mutant also displayed a marked reduction in survival in this infection model, similar to the highly attenuated *ras1∆* mutant strain^43^. Importantly, the reduction in *hda1∆* virulence was observed at both 30^o^C and 37^o^C, indicating that the thermotolerance defect alone cannot account for this strain’s hypovirulence (Fig. 5B,C). There was also a significantly reduced fungal burden for this strain at 4 days post-infection at both temperatures, consistent with its dramatic reduction in overall virulence (Fig. 5D,E).

Given that the *hda1Δ* mutant displayed the most striking virulence attenuation among the HDAC mutants, we also tested this strain in the murine inhalation model of cryptococcosis. We intranasally inoculated female C57BL/6 mice with the *hda1Δ* mutant strain, as well as isogenic reconstituted and wild-type strains. During the course of infection, we serially assessed surrogate end-points of progressive infection known to correlate with impaired survival (weight loss, neurological symptoms, and inability to maintain self-care)^44^. In a subset of infected mice, the lungs were prospectively assessed by quantitative fungal culture and histopathology at days 7 and 14 post inoculation.

**Figure 6:**
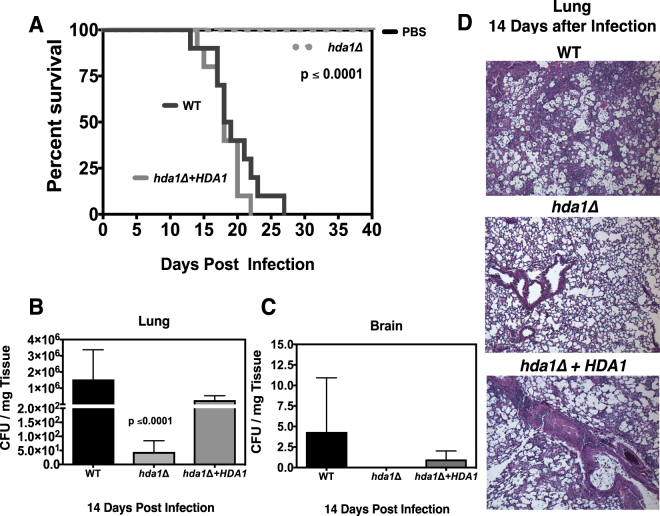
Effect of the *hda1Δ* mutant strain on virulence as assessed by the murine inhalational model of infection. **(A)** Ten C57BL/6 female mice were intranasally inoculated with the wild-type, *hda1Δ*, or *hda1∆* + *HDA1* strain to establish an infection. Animal survival was monitored for 40 days. Statistical test: Kaplan-Meier method. *p* < 0.0001 **(B)** Lungs and **(C)** brains of infected animals were harvested 14 days post infection, and quantitative cultures per mg tissue were performed. Statistical test: One-way ANOVA with Dunnett’s posttest. **(D)** Histopathological analysis of H&E-stained lung tissue from animals infected with the indicated strains after 14 days of infection.

Mice infected with the wild-type and the reconstituted strain exhibited a median survival time of 18 days, and a maximal survival time of 26 days (Fig. 6A). In contrast, all mice infected with the *hda1Δ* strain survived to the end of the experiment at 40 days with no signs of clinical illness (Fig. 6A). The number of CFUs recovered from the lungs and brains of infected mice was significantly reduced for the *hda1Δ* mutant compared to wild-type (Fig. 6B,C and S6). Additionally, at both time points, histopathological assessment with H&E staining showed fewer fungal cells and only minimal lung inflammation for the animals infected with the *hda1Δ* mutant (Fig. 6D and S6). Overall, these observations corroborate the macrophage and *G*. *mellonella* assays, reinforcing the importance of *Hda1* for virulence of *C*. *neoformans*.

### *Hda1* transcriptionally regulates genes required for adaptation and virulence

To identify the genes and processes regulated by *Hda1*, we used RNA-Seq to perform transcriptome analysis of the *hda1Δ* mutant compared to wild-type. For these experiments, we specifically chose incubation conditions (30 °C in minimal medium for 2 hours) to approximate those used for capsule and melanin induction as well as to match those used to measure HDAC gene transcript levels by RT-PCR.

**Figure 7:**
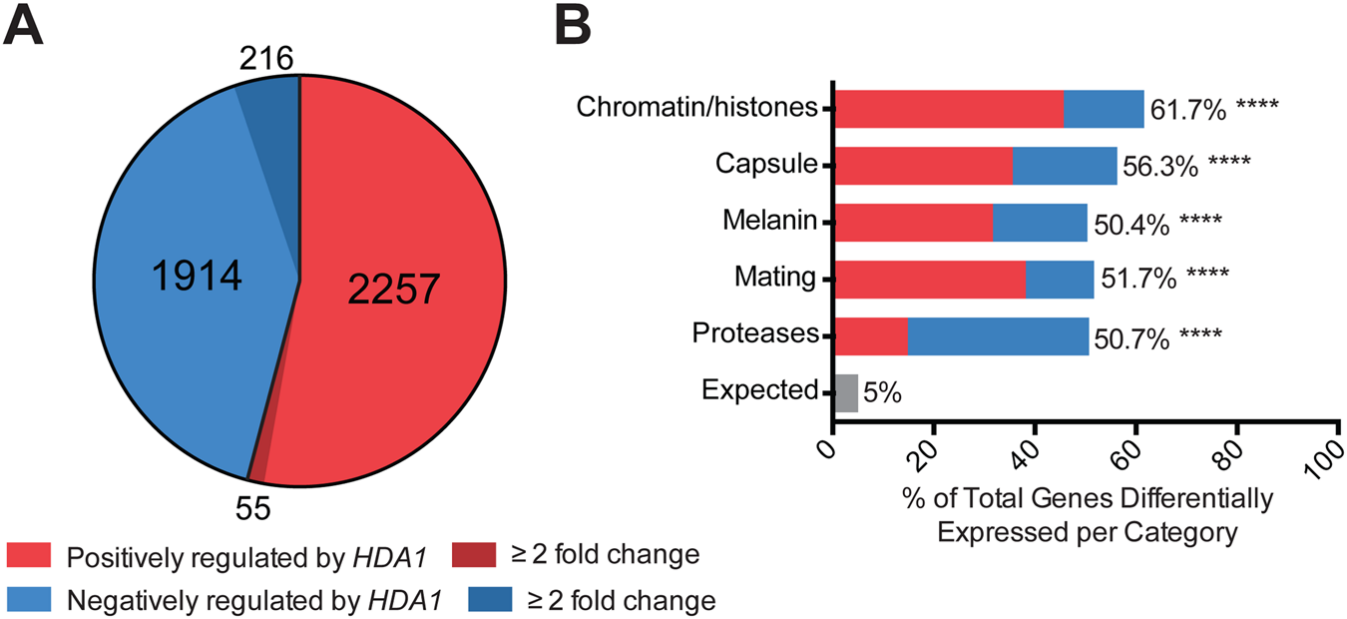
The transcriptional signature of the *hda1∆* mutant versus wild-type. **(A)** Hda1-dependent upregulated and downregulated genes in the *hda1∆* mutant versus wild-type as determined by two independent RNA-Seq analysis pipelines. Graph represents values from STAR/DeSeq2 analysis. **(B)** Modified gene ontology enrichment analysis of Hda1-dependent genes. The percent of genes differentially expressed in the *hda1∆* strain versus wild-type in each category is plotted on the x-axis. Colors represent the proportion of differentially expressed genes in each category positively or negatively regulated by *HDA1*. Statistical test: Chi-square of observed versus expected frequency of 5%; ****p < 0.0001.

We used two independent methods of data analysis to ensure reproducibility of results. Using the TopHat2/Cufflinks analysis pipeline^45,46^, we found a large number of genes (4715) with a statistically significant difference in transcript abundance between the wild-type and *hda1Δ* mutant strains (Fig. S7). Using the Star/DESeq2 analysis method^47–49^, we documented a similar number of genes (4982) with a statistically significant difference in transcript levels between these strains (Fig. S7B). These data sets were highly correlated, with 4171 genes found to differ in transcript abundance by both methods (Fig. S7C). Of these 4171 genes, 1914 were negatively regulated by *HDA1* (displayed increased mRNA levels in the *hda1∆* mutant) and 2257 were positively regulated by *HDA1* (displayed decreased mRNA levels in the *hda1∆* strain) (Fig. 7A). Although statistically significant, most of these changes in transcript levels were small in absolute magnitude, with only 271 genes demonstrating a transcriptional difference between the two strains of greater than 2-fold (216 genes negatively regulated by *HDA1*, and 55 genes positively regulated by *HDA1*).

We used the FungiDB database to perform a species-specific, modified gene ontology enrichment analysis (see Methods) to explore *HDA1*-dependent transcription of genes associated with cellular processes known to be affected by Hda1 protein function. These data are graphically represented in Fig. 7B, demonstrating notable enrichment of each functional category among the Hda1-regulated genes. There was a highly significant association between Hda1-regulation and genes involved in capsule, melanin, and mating, consistent with the observed *hda1Δ* mutant phenotypes. Importantly, the gene category most enriched among Hda1-dependent genes are those involved in chromatin/histone function **(**Fig. 7B**)**.

Consistent with the *hda1Δ* mutant defect in melanin production, the *LAC1* and *LAC2* transcript levels, corresponding to two laccase genes encoding the main phenoloxidases required for melanin production, were both reduced in the *hda1Δ* mutant. Similarly, multiple genes involved in various aspects of encapsulation were also expressed at lower levels in the *hda1Δ* strain compared to wild-type (Table 2 and Fig. 7B). Interestingly, most of these capsule-associated genes were only marginally altered in expression. This observation suggests that the altered phenotypes of the *hda1Δ* mutant were due to a cumulative effect on the expression of multiple genes rather than a single gene. Additionally, the similarity in transcriptional changes in multiple capsule-associated genes suggests some degree of HDAC-mediated transcriptional co-regulation for genes involved in this very important cellular process.

**Table 2:** Expression data for selected Hda1-regulated genes identified by modified gene ontology enrichment analysis.

Gene ID	Gene name	Product Description	log_2_(fold change)
Chromatin/Histones			
CNAG_00051	*SNT1*	Putative Set3c deacetylase complex subunit	−0.276468546
CNAG_00063		Histone H3	−0.466103457
CNAG_00085	*ASF1*	Histone chaperone *ASF1*	0.224363607
CNAG_00375	*GCN5*	Saga complex histone acetyltransferase	−0.338704771
CNAG_00561		Histone acetyltransferase type B catalytic subunit	−0.237158105
**CNAG_00660**	***HOS3***	**Histone deacetylase** ***HOS3***	**−0.576126726**
CNAG_00718	*CAC2*	Chromatin assembly factor 1 subunit B	−0.137046165
CNAG_00740	*SNF5*	Swi/Snf chromatin-remodeling complex subunit	−0.374848103
CNAG_01013		Chromatin binding protein	−0.66158404
CNAG_01018		Histone-lysine N-methyltransferase Su(var)3–9	−0.225072362
CNAG_01148	*FPR3*	Peptidyl-prolyl cis-trans isomerase	−0.664323021
CNAG_01520		Histone-arginine methyltransferase CARM1	−0.507582078
**CNAG_01563**	***HDA1***	**Histone deacetylase**	**−3.811428692**
CNAG_01648		Histone H4	−0.45507281
**CNAG_01699**	***CLR61***	**Histone deacetylase**	**−0.160504318**
CNAG_01863	*SNF2*	Chromatin remodeling complex ATPase	−0.202397993
CNAG_01972	*TAF10*	C2H2 zinc finger protein Zas1A	0.151175426
CNAG_02195		Origin recognition complex subunit 1	−0.746619743
CNAG_02215	*HAP3*	Transcriptional activator	−0.184025245
CNAG_02536	*TAF6*	Transcription initiation factor TFIID subunit 6	−0.1596263
CNAG_02749		Histone-lysine N-methyltransferase SUV420H	−0.450115461
CNAG_03188	*SET202*	Histone-lysine N-methyltransferase, H3 lysine-36 specific	−0.252801765
CNAG_03203		DNA polymerase epsilon p12 subunit	0.371759788
CNAG_04168		Histone H1/5	−0.582447791
**CNAG_05096**	***HOS1***	**Histone deacetylase**	**−0.123187881**
CNAG_05221		Histone H2A.Z	−0.183930793
**CNAG_05276**	***CLR62***	**Histone deacetylase**	**−0.176270846**
CNAG_05290	*SPT3*	Transcription initiation protein *SPT3*	0.159839958
CNAG_05404		Histone-lysine N-methyltransferase SUV39H	0.135941792
CNAG_05428	*TAF5*	Transcription initiation factor TFIID subunit 5	0.16375121
CNAG_06283	*LIV4*	Putative myb-like mRNA polymerase I termination factor	−0.479281412
CNAG_06392	*SGF29*	Putative saga histone acetyltransferase complex component	−0.489834833
CNAG_06544		Non-histone chromosomal protein 6	−0.61516928
CNAG_06597	*SPT8*	Transcriptional activator *SPT8*	−0.224863088
CNAG_06745		Histone H3	−0.576382711
CNAG_06746		Histone H2B	−0.606684422
CNAG_06747		Histone H2A	−0.53480499
CNAG_07027	*SPT2*	Protein *SPT2*	0.101538608
CNAG_07565	*TAF9*	Transcription initiation factor TFIID subunit 9B	0.194617264
CNAG_07572	*ELP3*	Pol II transcription elongation factor	−0.32202168
CNAG_07680	*HAP5*	Transcriptional activator *HAP5*	0.131411312
CNAG_07807		Histone H4	−0.37405091
Capsule			
CNAG_00600	*CAP60*	Capsule-associated protein	0.472665779
CNAG_00697	*UGE1*	UDP-glucose epimerase	0.406541427
CNAG_00701	*CAS31*	Protein involved in gxm O-acetylation	0.195896704
CNAG_00721	*CAP59*	Alpha-1,3-mannosyltransferase	−0.283339391
CNAG_02581	*CAS33*	Hypothetical protein	−0.230488663
CNAG_02805	*CAN1*	Carbonic anhydrase	0.381708027
CNAG_02885	*CAP64*	Capsule-associated protein	−0.411533956
CNAG_03322	*UXS1*	UDP-glucuronic acid decarboxylase	−0.192984755
CNAG_03426	*GMT2*	GDP-mannose transporter 2	−1.301133952
CNAG_03644	*CAS3*	Hypothetical protein	−0.680904969
CNAG_03695	*CAS41*	Probable sugar phosphate/phosphate translocator; capsule biosynthetic protein	−0.289383393
CNAG_03735	*CAP4*	Hypothetical protein	−0.583727866
CNAG_03929	*CAS42*	Solute carrier family 35, member C2	−0.140514137
CNAG_04312	*MAN1*	Mannose-6-phosphate isomerase	−0.199177397
CNAG_05023	*CAS91*	Putative maltose o-acetyltransferase	−0.396029552
CNAG_05081	*PDE1*	Phosphodiesterase	−0.17141564
CNAG_05139	*UGT1*	Solute carrier family 35 (UDP-sugar transporter), member A1/2/3	0.135099801
CNAG_05144	*CAN2*	Carbonic anhydrase	−0.881416777
CNAG_05222	*NRG1*	Transcriptional regulator Nrg1	−0.265150815
CNAG_05431	*RIM101*	pH-response transcription factor PacC/Rim101	0.181521283
CNAG_05817	*GMT1*	GDP-mannose transporter 1	−0.2343137
CNAG_06016	*CAP6*	Hypothetical protein	0.139069648
CNAG_06524	*FRE3*	Ferric reductase	4.348573694
Melanin			
CNAG_02434	*ATX1*	Putative copper ion transporter	−0.164690738
CNAG_03464	*LAC2*	Laccase	−0.253171475
CNAG_03465	*LAC1*	Laccase	−0.509427708
CNAG_05081	*PDE1*	Phosphodiesterase, phosphodiesterase, variant	−0.17141564
CNAG_05465	*GIB2*	Guanine nucleotide-binding protein subunit beta-like protein	−0.316312202
CNAG_06415	*CCC2*	Cu2 -exporting ATPase	0.152106422
CNAG_06524	*FRE3*	Ferric reductase	4.348573694
CNAG_07701	*CTR2*	Putative copper ion transporter	0.528660759
Mating			
CNAG_00293	*RAS1*	Ras-like protein	−0.198864829
CNAG_01262	*GPB1*	Guanine nucleotide-binding protein subunit beta	−0.14439011
CNAG_01452	*MAT3*	Mat3 pheromone repeat protein	−0.31601691
CNAG_01730	*STE7*	MAP kinase kinase	−0.264935377
CNAG_02756	*CDC43*	Geranylgeranyltransferase-I beta subunit	−0.21364078
CNAG_02883	*RAC1*	Rho family protein	−0.428370761
CNAG_03938	*CPR2*	Pheromone a factor receptor	0.549162139
CNAG_04119	*ROM2*	Rho guanyl-nucleotide exchange factor	0.13250298
CNAG_04761	*RAS2*	Ras family protein	−0.779751921
CNAG_05465	*GIB2*	Guanine nucleotide-binding protein subunit beta-like protein	−0.316312202
CNAG_05866	*PRM1*	Putative plasma membrane fusion protein	1.12944946
CNAG_05925	*CDC3*	Septin ring protein	−0.208014522
CNAG_05970	*PAK1*	Ste/Ste20/PakA protein kinase	0.070285615
CNAG_06806	*ETF1alpha*	Electron transfer flavoprotein alpha subunit	−0.523135543
CNAG_06808	*STE3alpha*	Pheromone a factor receptor	−0.657125254
CNAG_06811	*RPL22alpha*	Large subunit ribosomal protein L22e	−0.406283818
CNAG_06812	*SPO14alpha*	Phospholipase D1	−0.323123919
CNAG_06813	*CAP1alpha*	Hypothetical protein	−0.701589794
CNAG_06980	*STE11alpha*	MAPKK kinase, Ste/Ste11 protein kinase	0.317591405
CNAG_07407	*MFalpha3*	Mating-type pheromone alpha	−0.974668369
CNAG_07409	*RPO41alpha*	DNA-directed mRNA polymerase, mitochondrial	−0.66659612
CNAG_07410	*CID1alpha*	Hypothetical protein	−0.197735218
CNAG_07507	*STE50*	Protein kinase regulator	−0.274868604
Protease			
CNAG_00581		Saccharopepsin	0.406541427
CNAG_01343		ATP-dependent Clp protease ATP-binding subunit ClpX	0.397082268
CNAG_01688		ATP-dependent metalloprotease	−0.454088763
CNAG_02239		26 S protease regulatory subunit 4	0.447325323
CNAG_02282		Carboxypeptidase A4	0.450060236
CNAG_03904		26 S protease regulatory subunit 6B	0.150163286
CNAG_04380		Peptidase	−0.391189814
CNAG_04635		Endopeptidase	0.225975854
CNAG_04666		26 S protease regulatory subunit 8	0.186381652
CNAG_04906		26 S protease regulatory subunit 10B	0.282517098
CNAG_05742	*STP1*	Putative site-2 protease	−0.354935589
CNAG_05872		Endopeptidase	0.437070994
CNAG_06153		26 S protease regulatory subunit 6A-B	0.401220915
CNAG_06410		ATP-dependent Clp endopeptidase, proteolytic subunit ClpP	−0.20806081
CNAG_07520		Endopeptidase	0.661884424
*HDA1* associated			
CNAG_05333		Hypothetical protein	3.16455427
CNAG_06874		HpcH/HpaI aldolase/citrate lyase	0.721327^†^
CNAG_07651		DEAD-box ATP-dependent RNA helicase 26	0.924457338

Log_2_(fold change) values for *hda1∆* versus wild-type from DESeq2 analysis. Table amended from full list of differentially expressed genes in each category (Table S1). Negative changes represent genes positively regulated by Hda1; positive changes represent genes negatively regulated by Hda1.

^†^log_2_(fold change) value obtained from TopHat2/Cuffdiff analysis.

Similar trends of reduction in transcript abundance were evident for families of genes involved in mating and extracellular protease production (Table 2 and Fig. 7B). Additionally, several genes involved in transcription and chromatin remodeling, including other HDAC genes and the SAGA complex histone acetyltransferase (*GCN5*), were differently expressed in the *hda1Δ* mutant (Table 2).

Furthermore, we analyzed transcript levels of genes described to be regulated by Hda1. Hda1 was previously identified as a chromatin-related protein component of the *C*. *neoformans* Polycomb system, responsible for repression of gene expression in subtelomeric domains by recognizing H3K27 histone methylation^26^. In that study, *HDA1* was associated with repression of the expression of the subtelomeric genes CNAG_05333, CNAG_06874 and CNAG_07651^26^. In our RNA-Seq analyses we found that the same group of genes displayed increased levels of mRNA in the *hda1Δ* mutant (Table 2), reinforcing the concept that fungal Hda1 orthologs play a role in the assembly of subtelomeric heterochromatin in fungi as diverse as the basidiomycete *C*. *neoformans* and the ascomycete *S*. *pombe*^22,50^.

## Discussion

Chromatin remodeling by directed histone protein acetylation provides a rapid means to regulate transcription in response to changing environmental signals. This type of efficient and precise control of gene expression potentially allows microbial pathogens to maintain remarkable phenotypic plasticity in order to adapt to the many stresses encountered in the infected host. In fungi such as *A*. *nidulans*, *C*. *albicans*, *S*. *cerevisiae* and *S*. *pombe*, histone deacetylation and associated chromatin changes have been shown to mediate diverse cellular processes, including those associated with host adaptation and virulence^18,20,21,24,51^.

In the present work, we report a comprehensive characterization of the roles of Class I/II HDACs in the expression of virulence attributes of the opportunistic pathogenic fungus *C*. *neoformans*. Seven sequence homologues of *S*. *cerevisiae* and *S*. *pombe* class I and II HDAC genes were found in the *C*. *neoformans* genome. This number is higher than that described for many other fungal species. It also appears that there are two paralogous genes encoding homologs of the Clr6 HDAC (designated *CLR61* and *CLR62*), corroborating the phylogenetic study of Nishida, *et al*.^52^. Our phenotypic studies of the respective paralog mutant strains suggest that these two genes have undergone some degree of neofunctionalization. For example, the *clr61∆* mutant showed no impaired virulence attributes in several surrogate models of fungal virulence. In fact, this strain was more efficient in surviving inside macrophages *in vitro* and in *G*. *mellonella* larvae. On the other hand, the *clr62∆* mutant was sensitive to high temperature and displayed reduced capsule compared to wild-type.

### HDAC genes control specific development- and virulence-associated phenotypes

Individual mutant strains were obtained for each HDAC gene, and mutant strain virulence phenotypes were characterized in comparison to wild-type. We observed a large amount of phenotypic heterogeneity among these mutants, suggesting that individual HDAC proteins help to control the expression of a defined and specific set of genes. For example, the *hda1Δ*, *clr62Δ* and *rpd3Δ* mutant strains showed a reduction in the ability to grow on solid medium in the presence of surface-stressing agents such as SDS and CFW. In the presence of Congo red, a cell wall stressor, the growth reduction was subtle. Interestingly, recent investigators^53^ developed a chemical-genomic profiling approach to identify *S*. *cerevisiae* genes that may be related to growth under cell wall stress conditions and observed that specific HAT and HDAC genes were relevant to this process. Liu and collaborators observed growth defects at 37 °C for *C*. *neoformans hos2Δ* and *rpd3Δ* mutants^25^. In our assays, we observed that the *rpd3Δ* mutant presented impaired growth under cell wall stress conditions in addition to temperature, suggesting that the *RPD3* gene plays a role in cell surface damage prevention.

We also demonstrated that *hda1Δ*, *clr62Δ* and *hos3Δ* strains displayed a significant reduction in the polysaccharide capsule expansion, while the *hos1Δ* mutant presented a larger capsule in comparison to the wild-type. These opposing effects on capsule formation by different *C*. *neoformans* HDAC genes suggest that histone acetylation might be a rapid and tunable way to precisely control encapsulation in response to environmental changes. This type of complex phenotypic regulation is plausible given the number of genes involved in this important physiological process^34,54–58^. Interestingly, the deletion of genes involved in histone acetylation (HATs) in *C*. *neoformans* also resulted in alterations in capsule size^56,59^. Even though HATs and HDACs exhibit opposite enzymatic function, these data together highlight the importance of the chromatin acetylation and deacetylation dynamics in the regulation of capsule expansion.

The *hda1Δ* mutant also displayed a subtle increase in the cell body volume. This observation is likely related to the increase in the G2/M cell population observed at 24 h of growth in minimal medium at 37 °C. The *S*. *pombe* Hda1 homolog is also involved cell cycle control^60^. Furthermore, the *C*. *neoformans hda1Δ* mutant shift in G2/M cell population phenocopied the results of treatment of the wild-type strain with the HDAC inhibitor sodium butyrate^35^.

We noticed a transient delay in melanin production for the *hda1Δ* and *hos2Δ* mutant strains. A melanization defect for the *hos2Δ* mutant had been previously reported^25^. It is possible that the delay in the melanin synthesis is related to the cell arrest in G2/M we observed for the *hda1Δ* mutant, since melanization in *C*. *neoformans* is influenced by cell density through a quorum sensing-related mechanism^61,62^. Altered expression of genes involved in the phenol oxidation step of melanin formation was also noted for the *hda1Δ* mutant strain.

The production of secreted proteases is an important virulence trait for *C*. *neoformans*, as they are involved in the growth and survival in the presence of antifungal drugs^63^ and in the invasion of the central nervous system^64^. We have shown that the *hda1Δ* and *hos2Δ* strains display compromised secreted protease activities and that the *HDA1* and *HOS2* genes seem to be redundant in the regulation of this phenotype. Also, the simultaneous deletion of both genes resulted in impaired thermotolerance (data not shown). In *U*. *maydis* these two genes act redundantly in regulating pathogenesis. Interestingly, in *C*. *albicans HDA1* and *HOS2* exert opposite roles in the control of morphogenesis^21^. We report for the first time that HDAC genes are necessary for full mating hyphae formation in *C*. *neoformans*, thus corroborating the data we obtained with chemical inhibitors of these enzymes^35^. The *C*. *neoformans* mating process involves a complex series of signaling and developmental events, including pheromone sensing, hyphal morphological transition, meiosis, and sporulation. Of note, pheromone gene expression was also altered in the *hda1∆* mutant strain, suggesting a molecular mechanism for the observed deficient mating phenotypes.

HDAC mutants were studied in several infection models, including insects, isolated macrophages, and mice. Although the *hda1Δ* mutant showed the most prominently altered virulence in all of the models, we also noticed an *in vivo* survival defect in *G*. *mellonella* for the *hos2Δ* and *rpd3Δ* mutants. This result confirms prior findings of altered virulence for these mutant strains^25^. Distinct from the *hda1Δ* mutant, we did not observe any significant differences in the number of yeast cells recovered from larvae infected with the *hos2Δ* and *rpd3Δ* mutants versus the wild-type strain. This finding suggests that these fungal strains mediate virulence by multiple means, including the expression of factors that mediate host damage, as well as direct proliferation *in vivo*.

### Class I/II HDACs function in the context of other epigenetic regulators

We hypothesized that Hda1 is involved in the adaptation and regulation of gene expression during infection. Sugiyama demonstrated that the *S*. *pombe* Hda1 homologue Clr3 takes part in a multiprotein complex involved in transcriptional silencing within telomeric regions^50^. Dumesic reported that *C*. *neoformans* Hda1/Clr3 co-precipitated with the Polycomb repressor complex and that, in the *hda1∆/clr3∆* mutant strain, three genes related to subtelomeric regions were upregulated by qRT-PCR analysis^26^. We have corroborated the observed Hda1-repression of these same genes in our RNA-Seq experiment with a newly created *hda1∆* mutant strain.

In addition to classical HDACs, other proteins participate in histone deacetylation and chromatin remodeling in fungi. Recently, Bouklas and collaborators^65^ evaluated the function of *C. neoformans SIR2*, a member of a large family of NAD^+^-dependent, non-classical HDACs collectively known as sirtuins^30^. The authors demonstrated that *SIR2* loss resulted in a slightly impaired microbial fitness, which was rescued by Sir2 agonists. Additionally, Arras, *et al*.^66^ identified and deleted five *C. neoformans* sirtuin genes, demonstrating that two of the five deletion strains revealed mutant phenotypes *in vitro*. They found that three sirtuin genes (*SIR2*, *HST3* and *HST4*) play a role in virulence *in vivo*, supporting the importance of chromatin remodeling in *C. neoformans* pathogenesis. Similar to these studies in non-classical HDACs, our targeted mutagenesis of *C. neoformans* Class I/II HDACs demonstrates that not all HDAC mutants regulate virulence-associated phenotypes *in vitro*. However, our data also suggest redundant or opposing roles for distinct HDACs for phenotypes such as encapsulation or melanin production. Therefore, having multiple HDACs may allow *C. neoformans* to precisely control the expression of specific virulence attributes. This type of fine control of gene expression is accomplished by HDACs that regulate *FLO11* expression in *S. cerevisiae*^24,67^.

**Figure 8:**
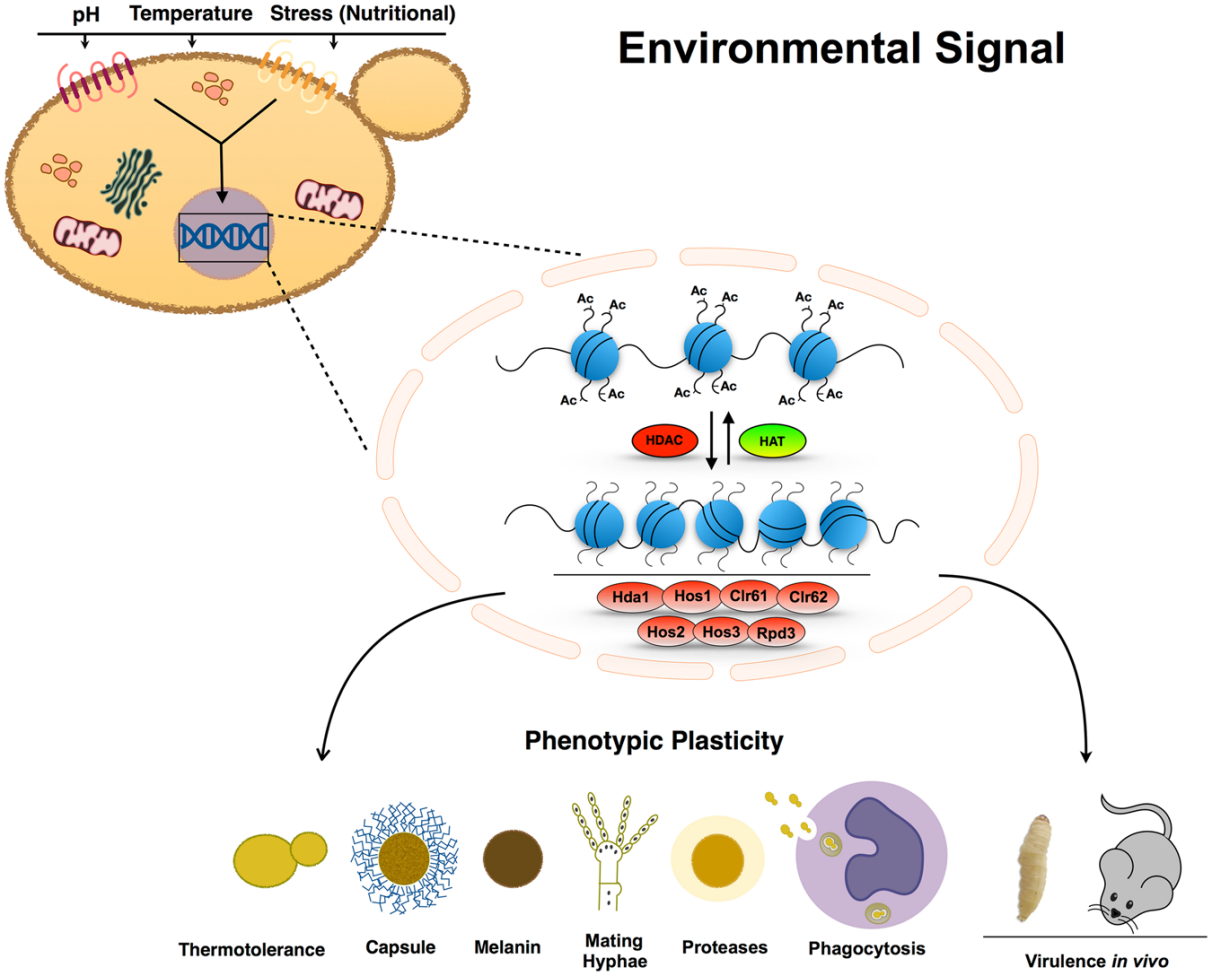
Model for HDAC regulation of the main virulence phenotypes in *C*. *neoformans*. Cells sense environmental cues (pH, high temperature, stress nutritional etc.) through membrane receptors and a signal is transmitted into the cell. The signal results in an adaptive response that requires chromatin remodeling mediated by the balance between HAT and HDAC activity. HDAC enzymes play a role in the regulation of the expression of all virulence phenotypes shown in the scheme. The loss of HDAC enzymes interferes with the correct adaptive response to environmental signals and impairs virulence factor expression.

In summary, we have shown that the Class I and II histone deacetylases play distinct and overlapping roles in *C*. *neoformans* virulence processes (Fig. 8). These processes include thermotolerance, capsule formation, melanin synthesis, protease activity and cell wall integrity. We also demonstrated that HDACs are necessary for *C*. *neoformans* survival in multiple models of cryptococcal infection (Fig. 8). Among the HDAC genes, *HDA1* controls multiple processes associated with fungal pathogenesis and development. The altered virulence of this mutant strain is likely due to its markedly reduced expression of capsule, melanin, and extracellular proteases, processes that are specifically required for microbial survival in the host. Finally, comparative transcriptional profiles of the *hda1Δ* and wild-type strains correlated mutant phenotypic changes with altered transcription of potentially relevant genes. The large number of genes with modest Hda1-dependent transcriptional changes suggest that the observed *hda1Δ* mutant phenotypes result from a complex and composite effect on the expression of multiple genes rather than a limited number of specific target genes.

Pharmacological manipulation of HDAC activity is being studied in several human diseases including malignancies and autoimmune diseases. For example, agents that control histone protein acetylation have been postulated as adjunctive therapy in HIV infection, potentially driving the virus out of latency and clearing infected patients of long-lived cellular reservoirs of infection^68^. In a similar manner, our data may contribute to the development of new therapeutic approaches for the treatment of fungal infections such as cryptococcosis.

## Materials and Methods

### Phylogenetic analysis of HDAC class I and II proteins

The predicted protein sequences of previously identified histone deacetylases for *S*. *cerevisiae* (Sc), *S*. *pombe* (Sp), and *U*. *maydis* (Um) were obtained from two fungal genome databases: the Broad Institute Fungal Genome Initiative (https://www.broadinstitute.org/fungal-genome-initiative, accessed 01/2015) and FungiDB (http://fungidb.org/fungidb/). These sequences were compared with HDAC gene homologues in *C*. *neoformans* (Cn), indicated by boxes. Multiple sequence alignments and phylogenetic analyses were performed using MUSCLE 3.7 (Multiple Sequence Comparison by Log-expectation): http://phylogeny.lirmm.fr/phylo_cgi/one_task.cgi? Task_type = muscle. A phylogenetic tree was prepared using MEGA7^69^.

### Strains, media, and growth conditions

Gene loci and strains used in this study are listed in Supplementary Tables S2 and S3. All strains were generated in the *C*. *neoformans* var. *grubii* strain H99 and were cultured on YPD (yeast extract [2%], peptone [1%], dextrose [2%]), unless stated otherwise. Fungal strains were stored in 15% glycerol at −80 °C until use.

### Molecular biology

Gene deletion constructs were generated using PCR primer extension/overlap and biolistic transformation according to the double-joint PCR approach^45^ to create a deletion construct with a split neomycin (*NEO*) or nourseothricin (*NAT*) resistance marker to replace each genomic coding sequence of interest by homologous recombination, as previously described^58^. The flanking homologous regions of the deletion constructs were amplified using the primers listed in Table S4. All mutant strains were created from at least two independent transformations, and the transformants were selected in medium supplemented with the appropriate antibiotic (100 µg/ml NAT, 100 µg/ml NEO). Mutations were confirmed by PCR using primers directed against the 5′- and 3′-flanking regions to ensure precise replacement of the native locus with the mutant allele.

To confirm the previously documented association between Hos2 and Rpd3 with *C*. *neoformans* virulence and *in vitro* virulence-associated phenotypes, we created independent *hos2Δ* (FS10) and *rpd3Δ* (FS13) mutants based on those from the 2015 Madhani *Cryptococcus* mutant collection (Fungal Genetics Stock Center)^25^. First, the whole deletion cassette with the *NAT* resistance marker was amplified by PCR and introduced by biolistic transformation into the wild-type strain H99. Mutations among the transformants were confirmed by PCR. The *clr61Δ* mutant was obtained from the *C*. *neoformans* mutant library^25^, and the ORF deletion was confirmed by PCR using primers AA4491 + AA4492. Given the lack of discernible mutant phenotypes for the *clr61* strain, either *in vitro* or *in vivo*, we elected not to create an independent mutant strain for this gene.

To create the *hda1Δ* mutant in the mating type **a** (*MAT****a***) strain background, the *MAT****α**** hda1Δ* mutant was crossed with the KN99 *MAT****a*** wide-type strain on MS mating media. Spores were isolated by microdissection, and recombinant spores were identified by PCR and neomycin (NEO) resistance. The *hda1Δ/hos2Δ and hda1Δ/rpd3Δ* double mutants were created by crossing the *hda1Δ* MAT**a** with the MAT**α** *hos2Δ* or *rpd3Δ* strain on MS medium. We used similar mating methods (genetic crosses with strain KN99**a**, microdissection of spores, PCR confirmation of mutations) to create multiple *clr62*Δ, *hos1*Δ, and *hos3*Δ strains, to confirm the association between the specific HDAC gene mutation and the observed alterations of phenotypes *in vitro*.

The *hda1Δ* + *HDA1* reconstituted strain was made by biolistically transforming the wildtype *HDA1* locus into the *hda1* mutant: the HDA1 locus was amplified using primers P11 (AAGG-AGATCT-GACACTTACGCTCTTT) and P12 (GTAG-TCTAGA-AGTTGTGTTCATCAGTCA) (*Bgl*II site underlined) for cloning into the *BamHI* site of the pCH233 plasmid containing the nourseothricin-resistance (*NAT*) marker.

### Capsule Induction and Quantitation of Capsule Size

Capsules were visualized by negative staining with India ink. Capsule radius was calculated^35^ using *ImageJ* (Fiji) software^70^. At least 100 cells were measured for each assay, and the data were presented by relative quantification (mutant/wild-type capsule size). The packed cell volume (as a surrogate measure of capsule size) was calculated as previously described^71,72^.

### Melanin assays

Melanin production was assessed by inoculation of the cells in a chemically defined minimal medium: 15 mM dextrose, 10 mM MgSO4, 29.4 mM KH2PO4, 13 mM glycine and 3 μM thiamine, pH 5.5, supplemented with 1 mM L-DOPA (Sigma-Aldrich) and incubated at 30 °C for 24 hours with shaking at 150 rpm. Melanization also was assessed on Niger seed agar^72^.

### Protease activity

Each strain (10^5^ cells) was inoculated on BSA agar medium (2% agar, 1% YNB medium, 1% BSA, 2% glucose) and incubated at 30 °C. The presence of a halo of clearance surrounding the colony was measured. The halo size was defined as the difference between (halo + colony diameter) minus (colony diameter). The diameter ratio (mutant halo size/wild-type halo size) was used for normalization of the data.

### Macrophage assay

We measured the ability of the fungal cells to survive macrophage ingestion according to previous protocols^73,74^. J774 A.1 cells (5 × 10^4^/well) were added to 96-well plates and activated by addition of 10 nM phorbol myristate acetate (PMA) and incubated for 1 h at 37 °C with 5% CO_2._ *C*. *neoformans* cells were opsonized with antibody 18B7 and added to the macrophages at a multiplicity of infection (MOI) of 1:1 (yeasts: macrophages), and the plates were incubated for 1 h at 37 °C in 5% CO_2_. The co-cultures were then washed three times with PBS to remove yeasts that were not internalized, and the plate was incubated at 37 °C in 5% CO_2_ for 18 h. Macrophages were lysed, and equal aliquots from each condition were quantitatively cultured on YPD agar and incubated at 30 °C for 2 days. Phagocytosis efficiency was measured for each strain, as previously described^75^, to ensure that altered engulfment did not account for differences in fungal recovery rates from this assay.

### Virulence assessment in the *G*. *mellonella* model

*G*. *mellonella* larvae were infected (5 × 10^4^ CFU) on the last left leg as previously described^43^. After infection, the caterpillars were placed at 30 or 37 °C, and monitored every 24 hours for a total of 10 days. After 4 days of infection, the 5 larvae pre-selected for CFU analysis were sacrificed, and hemolymph was quantitatively cultured on YPD + chloramphenicol (1 mg/ml).

### Virulence assessment in the murine inhalation model of cryptococcosis

The virulence of the *C*. *neoformans* strains was assessed using the murine inhalation model of cryptococcosis^44^. Briefly, groups of 10 female C57BL/6 mice were anesthetized with isoflurane and inoculated intranasally with 1 × 10^5^ fungal cells (in 25 μL sterile saline). The mice were monitored and sacrificed based on predetermined clinical endpoints that predict imminent mortality, using CO_2_ and bilateral thoracotomy. All studies were performed in compliance with American Veterinary Medical Association and Duke University institutional guidelines for animal experimentation.

### Histopathologic analysis and brain and lung fungal burden

Five additional female C57BL/6 mice per strain, per time point, were infected as described above and sacrificed at seven and fourteen days post-infection. One lung from each mouse was inflated and harvested in 10% neutral buffered formalin at the indicated days post-infection. All lungs were then embedded in paraffin, cut into 5 μm thick slices, and stained with H&E by the Duke University Histopathology Core Facility. For CFU analysis, the remaining lung and brains were removed surgically, weighed and then macerated in PBS. Colonies were counted and normalized by the weight of each tissue; the mean of the replicates for each individual were used to calculate the fungal burden in each organ.

### RNA preparation, sequencing and analysis

Three biological replicates of wild-type and *hda1Δ* mutant cells were incubated in YPD medium to mid-log phase and transferred to minimal medium for 2 h at 30 °C. RNA was extracted by using the Qiagen RNeasy Plant Minikit (Qiagen, Valencia, CA). Library preparation and RNA sequencing were performed by the Duke Sequencing and Genomic Technologies Shared Resource. Sequencing was performed on an Illumina HiSeq 2000/2500 instrument with 50 bp single end reads. To achieve sufficient sequence coverage for a reference transcriptome, the WT sample was sequenced with 72-bp paired-end reads. All raw and processed data have been deposited in NCBI’s Gene Expression Omnibus^76^ and are accessible through GEO Series accession number GSE109582 (https://www.ncbi.nlm.nih.gov/geo/query/acc.cgi?acc = GSE109582).

Data was analyzed by two independent methods to ensure rigor and reproducibility of the results. All reads were mapped to the *C*. *neoformans* reference genome using Tophat2 software^45^, and transcript quantitation was calculated via the Cufflinks/Cuffdiff pipeline^46^ using default parameters and a false discovery rate (FDR) of 10%. The *C*. *neoformans* H99 reference genome was obtained from the Sequencing Project at the Broad Institute of MIT and Harvard (accessed 03/31/2015). We excluded from the final analysis a limited number of genes (309) whose expression was unable to be interpreted either due to very low expression, ambiguous attribution of read assignments, or insufficient coverage. The annotation of gene function and location in this study was performed using FungiDB^77–79^.

To confirm and update our original analysis, a second independent alignment and differential expression analysis was performed following an RNA-Seq Bioconductor workflow^48^. This workflow utilizes STAR alignment software^49^ and the DESeq2 differential gene expression analysis package for R^47^ (FDR 10%). The *C*. *neoformans* strain H99 genome used for this analysis was obtained from NCBI (accessed July 2017). Similar to above, we excluded from our data analysis a limited number of genes (209) whose expression was unable to be interpreted either due to very low expression, ambiguous attribution of read assignments, or insufficient coverage.

Those genes that were significantly differentially regulated by both methods (4171), were analyzed by a modified gene ontology enrichment analysis using the FungiDB database. We generated a list of *C*. *neoformans* H99 genes associated with each cellular function of interest by using the “Search” function on FungiDB. The terms used in each search were “Chromatin”, “Histones”, “Capsule”, “Melanin”, “Mating”, and “Protease”. The lists generated were based on protein product descriptions (InterPro domains), user comments, PubMed citations, and phenotypic data included in the FungiDB database. Each functional gene list was compared to the significantly differentially regulated genes. The proportion/percent of significantly differentially regulated genes in each group was calculated, and enrichment was determined by a Chi-square test with a 5% expected frequency.

### Statistical analysis

Data are expressed as means ± SE of at least triplicate samples. Statistical analysis and significance were performed by using the GraphPad Prism version 6.01 for Mac (*GraphPad Software*) or R version 3.4.0 for Mac, and considered significant if *p*-values were <0.05. Normality and variance assumptions were verified using the *Shapiro-Wilk* test. One-way ANOVA and Dunnett’s posttest was applied to compare variation related to the control wild-type. Two-way ANOVA and Tukey’s posttest were used to compare different groups with more than one variable. The 95% confidence interval was determined for all the experiments.

### Ethics statement

All animal experimentation was performed according to established protocols approved by the Duke University Institutional Animal Care and Use Committee (IACUC). The minimum number of animals was used for each experiment to ensure statistical significance based pre-test predictions. All infections were performed after inhalational anesthesia with isoflurane. Daily animal care was overseen by Duke Vivarium veterinary staff. According to IACUC-approved protocols, animals were sacrificed by CO_2_ asphyxiation, followed by a secondary means of ensuring animal death (bilateral thoracotomy), according to institutional guidelines.

### Data Availability

All data generated or analyzed during this study are included in this published article (or its Supplementary Information files). All microbiological strains, plasmids and sequencing data files are available to the scientific community upon request.

## Electronic supplementary material

Supplementary information information
