# Supplementary material for: HDAC genes play distinct and redundant roles in Cryptococcus neoformans virulence
Source: Sci Rep. 2018 Mar 26;8:5209. doi: 10.1038/s41598-018-21965-y (PMC5979944; doi:10.1038/s41598-018-21965-y)
Supplement: Supplementary file 1 — Supplementary information information [file 41598_2018_21965_MOESM1_ESM.docx]

**Supplementary Material**

Brandao, et al., 2018 (HDAC genes play distinct and redundant roles in *Cryptococcus neoformans* virulence)

**Supplementary Methods**

**RNA extraction and RT-qPCR**

The wild-type strain was grown for 24 h in YPD at 30 ºC. Cells were washed three times and counted, and cell density was adjusted to 1 x 10^8^ cells/ml in minimal medium. The cells were incubated at 30°C, and collected by centrifugation at times 0, 30, 60 and 180 minutes. The cell pellet was resuspended in 1 ml of Trizol reagent (Invitrogen) and subjected to mechanical lysis with glass beads of 0.5-mm for 20 min by vortexing. After lysis, the total RNA was extracted according to Trizol instructions, with some modifications: centrifugation at 12,000g / 20 min/ 2 °C; the precipitation stage with isopropanol was carried out at -20 °C for 1 h, followed by centrifugation at 16,000g / 20min / 2 ºC. The RNA samples were quantified by NanoDropTM and the integrity was verified by gel electrophoretic analysis 1% agarose. The cDNA synthesis was performed using 2 μg of total RNA, using High-Capacity cDNA Reverse Transcription Kits (Applied Biosystems). The amplifications, using cDNA as template for the quantitative analysis of transcripts, were performed using the Fast SYBR Green Master Mix Kit (Applied Biosystems) according to the manufacturer's instructions. The method for relative quantification based on the 2^−ΔΔCT^ ^1^ was used. To avoid amplification from possible contaminant DNA sequences, the primers were designed in the exon-exon junction (**Table S3**). The actin-encoding gene *ACT1* was used as a control for normalization, and the zero time samples were used as calibrators.

**Cell Cycle Analysis**

The *hda1*Δ, wild-type and reconstituted (*hda1* + *HDA1*) strains were grown in minimal medium at 37 ºC/150 rpm for 24 h. The yeasts were centrifuged (1000 *g* / 5 minutes) and resuspended in 1x NS buffer (10 ml 1M Tris-HCL pH 7.5, sucrose 85.6 g, 2 ml 0.5M EDTA pH 8, 0.095g MgCl2, 0147g CaCl 2, 0.0136 g ZnCl 2, 0.096 phenylmethylsulfonyl fluoride, 0.49 ml of 2-mercaptoethanol, 989 ml H_2_Od) and centrifuged again for 30s. Resuspension was carried out in 200 μL of Master Mix solution (180 μL 1x NS, 14 μL RNase [15 μg / μl ], 6 μl propionate Iodide-PI [1 μg / μl ]) and incubated at room temperature for 2 h. A 50 μL aliquot of the cell suspension was added to 500 μL of Tris-PI solution (428 μl 1M Tris pH 7.5, 18 μl PI [1 μg / μl ]), incubated on ice and transported to the Duke Cancer Institute Flow Cytometry Shared Resource for Linear scale analysis (BD FACS Calibur ™). The cell populations were separated according to the stages of the cell cycle (G1/G0, S or G2/M). For each condition, 10.000 events were measured. Data were analyzed using *FlowJo* software version 7.6.5 for Mac.

**RNA-Seq data analysis**

Volcano plots of the log_2_(fold change) versus –log_10_(pvalue) for each data set were generated in R (R version 3.4.0 for MacOS). A venn diagram of the genes differentially expressed by both methods was generated in R using the Vennerable package ^2^ and gene IDs (CNAG numbers) as inputs.

**Supplementary References**

1 Livak, K. J. & Schmittgen, T. D. Analysis of relative gene expression data using real-time quantitative PCR and the 2(-Delta Delta C(T)) Method. *Methods* **25**, 402-408, doi:10.1006/meth.2001.1262 (2001).

2 Wilkinson, L. Exact and approximate area-proportional circular Venn and Euler diagrams. *IEEE Trans Vis Comput Graph* **18**, 321-331, doi:10.1109/TVCG.2011.56 (2012).

3 Mylonakis, E. *et al.* *Galleria mellonella* as a model system to study *Cryptococcus neoformans* pathogenesis. *Infection and immunity* **73**, 3842-3850, doi:10.1128/IAI.73.7.3842-3850.2005 (2005).

4 Perfect, J. R., Lang, S. D. & Durack, D. T. Chronic cryptococcal meningitis: a new experimental model in rabbits. *The American journal of pathology* **101**, 177-194 (1980).

5 Nielsen, K. *et al.* Sexual cycle of *Cryptococcus neoformans* var. *grubii* and virulence of congenic a and alpha isolates. *Infection and immunity* **71**, 4831-4841 (2003).

6 Alspaugh, J. A., Cavallo, L. M., Perfect, J. R. & Heitman, J. RAS1 regulates filamentation, mating and growth at high temperature of *Cryptococcus neoformans*. *Mol Microbiol* **36**, 352-365 (2000).

**Supplementary Figures**

**Supplementary Figure S1**.

**Supplementary Figure S1**. **Real-time PCR confirms expression and transcriptional induction of *C. neoformans* HDAC genes.** Wild-type *C. neoformans* wild-type cells were incubated in YPD medium to mid-logarithmic growth phase, and transferred to minimal medium for 30, 60 and 180 minutes. RNA was isolated, and quantitative real-time PCR was used to assess the relative transcript levels for each HDAC gene at each time point, relative to expression at time zero. Error bars represent standard deviations for three technical replicates in two separate experiments. Statistical analysis was performed using *two-way* ANOVA and Dunnett’s posttest.

**Supplementary Figure S2**.


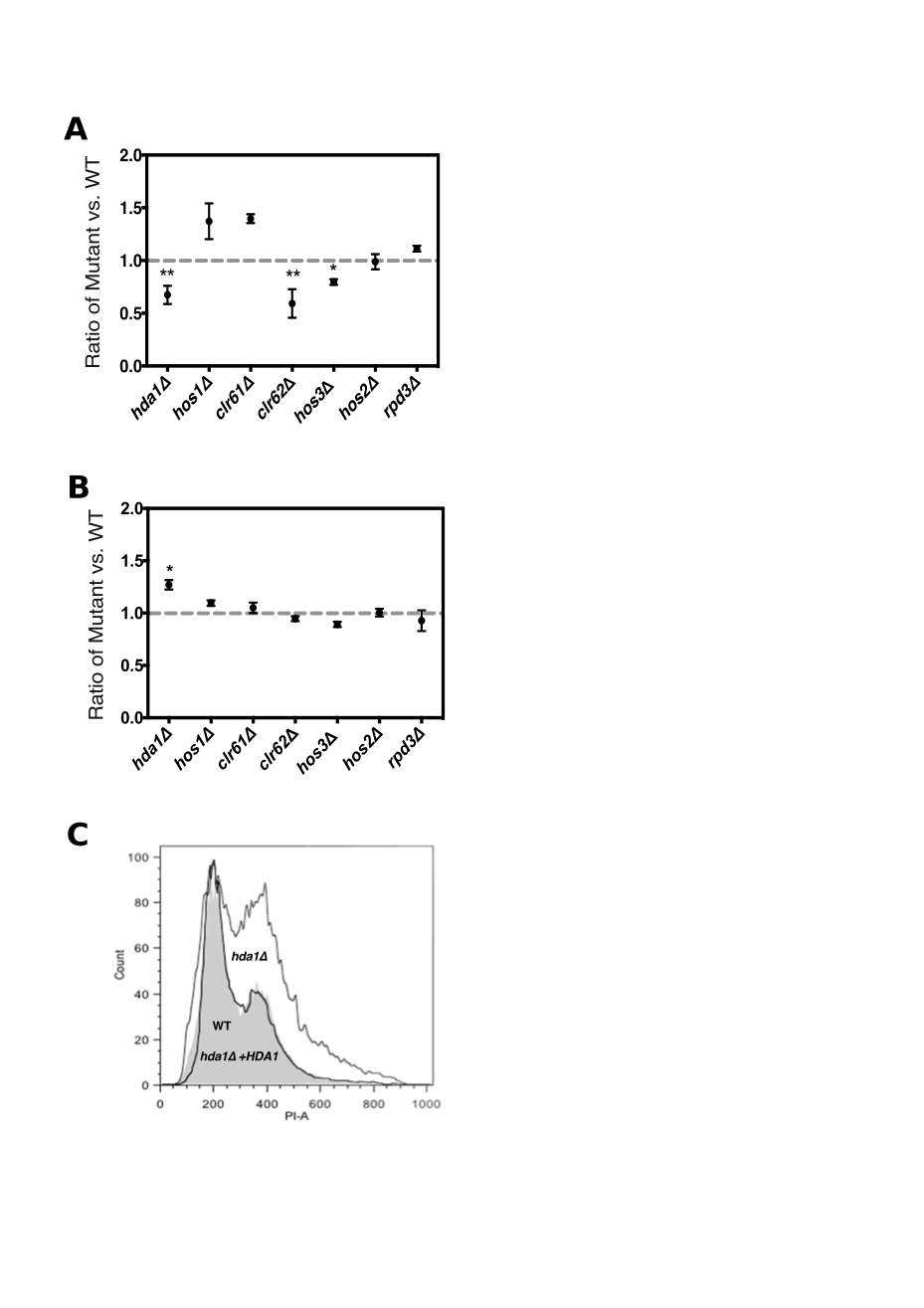


**Supplementary Figure S2**. **HDAC genes control encapsulation and cell cycle progression**. To quantify capsule and cell size changes, each strain was incubated in CO_2_-independent tissue culture medium for 72 hours to induce capsule formation. (**Α**) Capsule and cell diameter were independently assessed by determining packed cell volume for normalized cell suspensions in micro-capillary tubes. Data are represented as the ratio of average packed cell volume for each mutant strain compared to that of wild-type (red dashed line). Error bars represent standard errors of the means. (**B**) Average cell body size was measured in 100 cells; the capsule size was calculated as the difference between the cell diameter and the diameter of the cell body (error bars = SE). Statistical test: *One-way ANOVA* with Dunnett’s post-test multiple-comparison test were used to compare the means of results from three replicates for three independent experiments. *** *p <* 0.001; ** *p <*0.01; * *p <* 0.0414. (**C**) **The *hda1Δ* mutant demonstrates a shift in cell population in G2 / M**. Cells were incubated in minimal medium (MM) for 24 h at 37 ºC, and DNA content was assessed by flow cytometry of propidium iodide-stained cells. The histogram shows overlap of cell populations from the wild-type (gray shading), *hda1Δ* mutant (gray line), and *hda1Δ + HDA1* reconstituted strain (black line).

**Supplementary Figure S3.**

**Supplementary Figure S3. HDACs and extracellular protease production**. Indicated strains were spotted onto BSA agar and incubated at 30 ºC for 3 days. The presence of a peripheral clear halo indicates protease activity. The radius of clearing was calculated as the difference between the halo diameter and the diameter of the colony. Data are represented as average ratio of each mutant strain normalized to the wild-type control (red dashed line). Error bars represent standard errors of the means. Statistical test: *One-way ANOVA* with Dunnett’s multiple-comparison test used to compare the means of results from three independent experiments. **** *p <* 0.0001; * *p <* 0.05

**Supplementary Figure S4.**

**Supplementary Figure S4. HDACs and mating.** The MAT**α** wild-type and HDAC mutants were incubated in mating mixtures with the wild-type MAT**a** strain KN99a on MS agar at room temperature, protected from the light. The edges of the mating mixtures were assessed for mating hyphae at 7 and 10 days (100x).

**Supplementary Figure S5.**

**Supplementary Figure S5. HDACs and virulence in *Galleria mellonella*.** (**A-E**) The indicated strains were inoculated into the greater wax moth *G. mellonella*, and larval survival was monitored at 30 ^o^C. Mock infections with PBS injections were used as uninfected controls, and all infections were compared with the *ras1∆* hypovirulent mutant ^3^. Statistical test: Kaplan-Meier method. *p* < 0,0085; *p* < 0,0001. (**F**) In a separate experiment, five larvae from each group were assessed for fungal burden by quantitative culture on the fourth day post infection (CFU/caterpillar). Statistical test: *One-way ANOVA* with Dunnett’s posttest.

**Supplementary Figure S6.**

**Supplementary Figure S6. Effect of the *hda1***𝛥 **mutant strain on virulence as assessed by the murine model of infection.** (**A**) CFU analysis from infected mouse lungs at 7 days post infection. Infected mouse lungs were harvested and CFUs were assessed per mg of lung tissue. (**B**) Histopathological analysis revealed decreased yeast numbers and inflammatory cell infiltration in the lung of mice infected by *hda1∆* mutant. Lungs were harvested on day 7 post inoculation and H & E stained. Arrows mark *C. neoformans* cells. Statistical test: Student’s t-test*.*

**Supplementary Figure S7.**


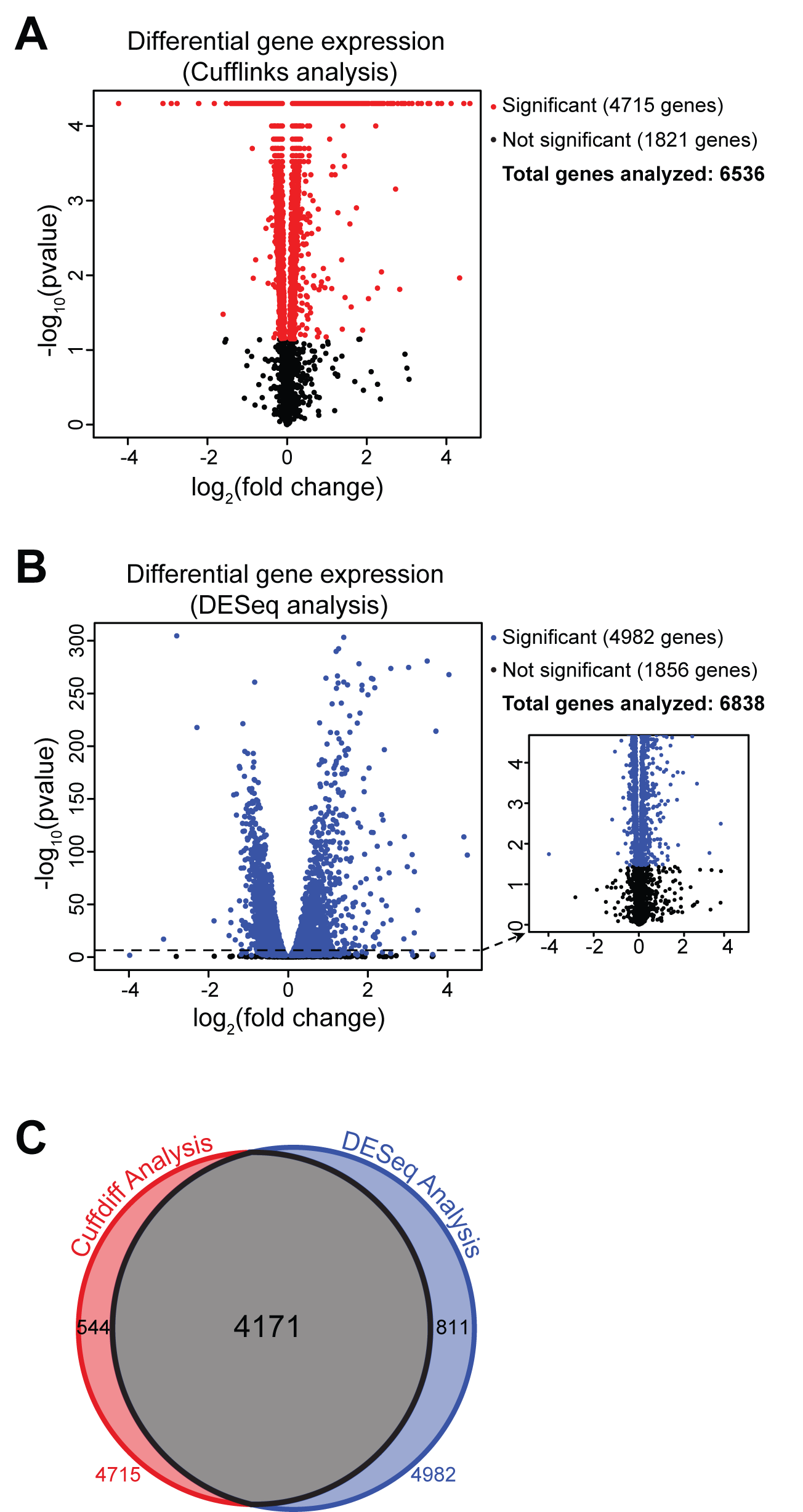


**Supplementary Figure S7.** **(A, B)** Volcano plots of log_2_(fold change) versus –log_10_(pvalue) of **(A)** Cufflinks and **(B)** DESeq2 differential expression analyses. Colored points represent significantly differentially expressed genes (p < 0.05). **(C)** Venn diagram of the number of overlapping significantly differentially expressed genes from Cufflinks and DESeq2 differential expression analyses.

**Supplementary Table 1.** Expression data for all genes identified by modified gene ontology enrichment analysis. Log_2_(fold change) values for *hda1∆* versus wild-type from DESeq2 analysis. Negative changes represent genes positively regulated by *HDA1*; positive changes represent genes negatively regulated by *HDA1*.

|  | Gene ID | Gene name | Product Description | log2(fold change) |
| --- | --- | --- | --- | --- |
| Chromatin/Histones | | | | |
|  | CNAG_00051 | *SNT1* | Putative Set3c deacetylase complex subunit | -0.276468546 |
|  | CNAG_00063 |  | Histone H3 | -0.466103457 |
|  | CNAG_00085 | *ASF1* | Histone chaperone *ASF1* | 0.224363607 |
|  | CNAG_00375 | *GCN5* | Saga complex histone acetyltransferase | -0.338704771 |
|  | CNAG_00561 |  | Histone acetyltransferase type B catalytic subunit | -0.237158105 |
|  | CNAG_00660 | *HOS3* | Histone deacetylase *HOS3* | -0.576126726 |
|  | CNAG_00677 |  | E3 ubiquitin-protein ligase UHRF1 | -0.837257226 |
|  | CNAG_00718 | *CAC2* | Chromatin assembly factor 1 subunit B | -0.137046165 |
|  | CNAG_00740 | *SNF5* | Swi/Snf chromatin-remodeling complex subunit | -0.374848103 |
|  | CNAG_01013 |  | Chromatin binding protein | -0.66158404 |
|  | CNAG_01018 |  | Histone-lysine N-methyltransferase Su(var)3-9 | -0.225072362 |
|  | CNAG_01148 | *FPR3* | Peptidyl-prolyl cis-trans isomerase | -0.664323021 |
|  | CNAG_01201 |  | Hypothetical protein | -0.211065231 |
|  | CNAG_01301 |  | Hypothetical protein | 0.200961659 |
|  | CNAG_01520 |  | Histone-arginine methyltransferase CARM1 | -0.507582078 |
|  | CNAG_01563 |  | Histone deacetylase 6/10 | -3.811428692 |
|  | CNAG_01600 | *RRB1* | Ribosome assembly protein *RRB1* | -0.607358709 |
|  | CNAG_01648 |  | Histone H4 | -0.45507281 |
|  | CNAG_01699 |  | Histone deacetylase 1/2 | -0.160504318 |
|  | CNAG_01863 | *SNF2* | Chromatin remodeling complex ATPase | -0.202397993 |
|  | CNAG_01972 | *TAF10* | C2H2 zinc finger protein Zas1A | 0.151175426 |
|  | CNAG_02034 |  | Hypothetical protein | -0.45241697 |
|  | CNAG_02195 |  | Origin recognition complex subunit 1 | -0.746619743 |
|  | CNAG_02215 | *HAP3* | Transcriptional activator | -0.184025245 |
|  | CNAG_02218 |  | Hypothetical protein | -0.134685492 |
|  | CNAG_02536 | *TAF6* | Transcription initiation factor TFIID subunit 6 | -0.1596263 |
|  | CNAG_02749 |  | Histone-lysine N-methyltransferase SUV420H | -0.450115461 |
|  | CNAG_03188 | *SET202* | Histone-lysine N-methyltransferase, H3 lysine-36 specific | -0.252801765 |
|  | CNAG_03203 |  | DNA polymerase epsilon p12 subunit | 0.371759788 |
|  | CNAG_03301 | *TIM13* | Mitochondrial import inner membrane translocase subunit *TIM13* | 0.105691735 |
|  | CNAG_04168 |  | Histone H1/5 | -0.582447791 |
|  | CNAG_04786 |  | Hypothetical protein | 0.191007937 |
|  | CNAG_04970 |  | Hypothetical protein | 0.40909776 |
|  | CNAG_05096 |  | Histone deacetylase 1/2 | -0.123187881 |
|  | CNAG_05221 |  | Histone H2A.Z | -0.183930793 |
|  | CNAG_05262 |  | Hypothetical protein | -0.342496439 |
|  | CNAG_05276 |  | Histone deacetylase 1/2 | -0.176270846 |
|  | CNAG_05290 | *SPT3* | Transcription initiation protein *SPT3* | 0.159839958 |
|  | CNAG_05404 |  | Histone-lysine N-methyltransferase SUV39H | 0.135941792 |
|  | CNAG_05423 |  | SET domain-containing protein | -0.405270396 |
|  | CNAG_05428 | *TAF5* | Transcription initiation factor TFIID subunit 5 | 0.16375121 |
|  | CNAG_05782 |  | Mortality factor 4-like protein 1 | -0.487126152 |
|  | CNAG_06283 | *LIV4* | Putative myb-like mRNA polymerase I termination factor | -0.479281412 |
|  | CNAG_06392 | *SGF29* | Putative saga histone acetyltransferase complex component | -0.489834833 |
|  | CNAG_06451 |  | Hypothetical protein | 0.174014015 |
|  | CNAG_06544 |  | Non-histone chromosomal protein 6 | -0.61516928 |
|  | CNAG_06597 | *SPT8* | Transcriptional activator *SPT8* | -0.224863088 |
|  | CNAG_06745 |  | Histone H3 | -0.576382711 |
|  | CNAG_06746 |  | Histone H2B | -0.606684422 |
|  | CNAG_06747 |  | Histone H2A | -0.53480499 |
|  | CNAG_07027 | *SPT2* | Protein *SPT2* | 0.101538608 |
|  | CNAG_07420 |  | Hypothetical protein | -0.149858323 |
|  | CNAG_07565 | *TAF9* | Transcription initiation factor TFIID subunit 9B | 0.194617264 |
|  | CNAG_07572 | *ELP3* | Pol II transcription elongation factor | -0.32202168 |
|  | CNAG_07680 | *HAP5* | Transcriptional activator *HAP5* | 0.131411312 |
|  | CNAG_07688 |  | Hypothetical protein | 0.326582659 |
|  | CNAG_07807 |  | Histone H4 | -0.37405091 |
|  | CNAG_07904 |  | Hypothetical protein | -0.275452709 |
| Capsule | | | | |
|  | CNAG_00025 | *VCX1* | Vacuolar calcium exchanger | -0.336166974 |
|  | CNAG_00051 | *SNT1* | Putative set3c deacetylase complex subunit | -0.276468546 |
|  | CNAG_00124 | *CAS32* | Hypothetical protein | -0.517621409 |
|  | CNAG_00130 | *RCK2* | Predicted protein kinase | 0.448163545 |
|  | CNAG_00193 | *GAT1* | Gata type zinc finger protein | -0.215402435 |
|  | CNAG_00268 | *ILV2* | Acetolactate synthase, mitochondrial | -0.504745534 |
|  | CNAG_00293 | *RAS1* | Ras-like protein | -0.198864829 |
|  | CNAG_00375 | *GCN5* | Saga complex histone acetyltransferase | -0.338704771 |
|  | CNAG_00415 | *CDC2801* | Cyclin-dependent kinase of PITSLRE subfamily | 0.280245912 |
|  | CNAG_00440 | *SSN801* | Cyclin subunit of mediator subcomplex | 0.260537496 |
|  | CNAG_00600 | *CAP60* | Capsule-associated protein | 0.472665779 |
|  | CNAG_00601 | *CEL1* | Putative glycosyl hydrolase | 0.217686372 |
|  | CNAG_00654 | *SRX1* | Sulfiredoxin | -0.468490799 |
|  | CNAG_00697 | *UGE1* | UDP-glucose epimerase | 0.406541427 |
|  | CNAG_00701 | *CAS31* | Protein involved in gxm O-acetylation | 0.195896704 |
|  | CNAG_00721 | *CAP59* | Alpha-1,3-mannosyltransferase | -0.283339391 |
|  | CNAG_00746 | *CAS35* | Hypothetical protein | 0.397082268 |
|  | CNAG_00757 | *DOA4* | Ubiquitin carboxyl-terminal hydrolase 8 | -0.454088763 |
|  | CNAG_00815 | *SIT1* | Siderophore iron transporter | 0.356398599 |
|  | CNAG_00979 | *CTR4* | Solute carrier family 31 (copper transporter) | 0.180535376 |
|  | CNAG_00986 | *UBA4* | Urm1 activating enzyme | 0.447325323 |
|  | CNAG_01163 | *RAD54* | DNA repair and recombination protein RAD54-like protein | 0.240280635 |
|  | CNAG_01172 | *PBX1* | Parallel beta-helix repeat protein | 0.450060236 |
|  | CNAG_01242 | *HAPX* | Hypothetical protein | -0.820609904 |
|  | CNAG_01283 | *CAP5* | Hypothetical protein | 0.115163906 |
|  | CNAG_01350 | *RMD5* | Putative ubiquitin ligase | 0.150163286 |
|  | CNAG_01537 | *VAD1* | Virulence associated ATP-dependent mRNA helicase | -0.391189814 |
|  | CNAG_01562 | *BLP4* | Pr4/barwin domain protein | -0.604397355 |
|  | CNAG_01611 | *LIV8* | Infection related protein of unknown function | 0.148936278 |
|  | CNAG_01644 | *MLN4* | Protein of unknown function | 0.225975854 |
|  | CNAG_01653 | *CIG1* | Cytokine inducing-glycoprotein | 0.186381652 |
|  | CNAG_01678 | *NHA1* | Na+/H+ antiporter | 0.282517098 |
|  | CNAG_02008 | *OVA1* | Protein of unknown function | 1.435589647 |
|  | CNAG_02071 | *BNI4* | Putative scaffold protein | -0.226897296 |
|  | CNAG_02215 | *HAP3* | Transcriptional activator | -0.354935589 |
|  | CNAG_02236 | *PPG1* | Type 2a-like serine/threonine-protein phosphatase | 0.437070994 |
|  | CNAG_02409 | *LIV5* | Protein of unknown function | 0.401220915 |
|  | CNAG_02579 |  | Hypothetical protein | -0.20806081 |
|  | CNAG_02581 | *CAS33* | Hypothetical protein | -0.230488663 |
|  | CNAG_02745 | *DCR2* | Hypothetical protein | 0.288214239 |
|  | CNAG_02753 | *LIV13* | Endoplasmic reticulum protein | 0.314545254 |
|  | CNAG_02771 |  | DNA repair and recombination protein RAD54B | 0.661884424 |
|  | CNAG_02797 | *CPL1* | Putative secreted protein | 0.201869216 |
|  | CNAG_02805 | *CAN1* | Carbonic anhydrase | 0.381708027 |
|  | CNAG_02810 | *PUF4* | Pumilio-family mRNA binding protein | -0.630085894 |
|  | CNAG_02827 | *RUB1* | Ubiquitin-like protein Nedd8 | -0.524049637 |
|  | CNAG_02885 | *CAP64* | Capsule-associated protein | -0.411533956 |
|  | CNAG_02930 | *LIV10* | Virulence related protein of unknown function | -0.20573052 |
|  | CNAG_03116 | *HCM1* | Forkhead transcription factor 3 | -0.21890327 |
|  | CNAG_03138 | *CUL3* | Ubiquitin-protein ligase | 0.086103217 |
|  | CNAG_03188 | *SET202* | Histone-lysine N-methyltransferase, H3 lysine-36 specific | -0.252801765 |
|  | CNAG_03322 | *UXS1* | UDP-glucuronic acid decarboxylase | -0.192984755 |
|  | CNAG_03345 | *SSD1* | Putative translational repressor | 0.408348255 |
|  | CNAG_03406 | *SPP101* | Putative compass/set1c complex subunit | -0.214246036 |
|  | CNAG_03409 | *SKN7* | Response regulator and transcription factor | 0.382459838 |
|  | CNAG_03426 | *GMT2* | GDP-mannose transporter 2 | -1.301133952 |
|  | CNAG_03464 | *LAC2* | Laccase | -0.253171475 |
|  | CNAG_03637 | *YKU80* | ATP-dependent DNA helicase 2 subunit 2 | 0.639234028 |
|  | CNAG_03644 | *CAS3* | Hypothetical protein | -0.680904969 |
|  | CNAG_03648 | *KRE5* | UDP-glucose:glycoprotein glucosyltransferase | 0.166237931 |
|  | CNAG_03670 | *IRE1* | Serine/threonine-protein kinase, unfolded protein response | -0.182448298 |
|  | CNAG_03680 | *ROM21* | Rho guanine nucleotide exchange factor | 0.359724017 |
|  | CNAG_03695 | *CAS41* | Probable sugar phosphate/phosphate translocator; capsule biosynthetic protein | -0.289383393 |
|  | CNAG_03716 | *BLP6* | Pr4/barwin domain protein | -1.554114441 |
|  | CNAG_03735 | *CAP4* | Hypothetical protein | -0.583727866 |
|  | CNAG_03872 | *UBP7* | Ubiquitin carboxyl-terminal hydrolase | 0.115163906 |
|  | CNAG_03894 | *PDR802* | Hypothetical protein | -0.984917006 |
|  | CNAG_03917 | *NUP75* | Nuclear pore complex protein Nup85 | -0.380589798 |
|  | CNAG_03929 | *CAS42* | Solute carrier family 35, member C2 | -0.140514137 |
|  | CNAG_04090 | *ATF1* | Activating transcription factor | 0.615132694 |
|  | CNAG_04119 | *ROM2* | Rho guanyl-nucleotide exchange factor | 0.13250298 |
|  | CNAG_04158 | *HIRA* | Protein *HIR1* | -0.264210018 |
|  | CNAG_04215 | *MET3* | Sulfate adenylyltransferase | -0.482042119 |
|  | CNAG_04312 | *MAN1* | Mannose-6-phosphate isomerase | -0.199177397 |
|  | CNAG_04352 | *ZAP103* | Zinc-finger protein | 0.152630637 |
|  | CNAG_04371 | *HSV2* | Hypothetical protein | 0.661634112 |
|  | CNAG_04552 | *ITR1A* | Putative inositol transporter | 2.989502587 |
|  | CNAG_04609 | *AGO1* | Argonaute protein | -0.18555876 |
|  | CNAG_04611 | *UBC8* | Ubiquitin-conjugating enzyme E2 H | 0.564493528 |
|  | CNAG_04678 | *YPK1* | Protein kinase | 0.112244349 |
|  | CNAG_04730 | *GPR4* | Hypothetical protein | -0.289720147 |
|  | CNAG_04755 | *BCK1* | Mitogen-activated protein (MAP) kinase kinase kinase | -0.328412101 |
|  | CNAG_04761 | *RAS2* | Ras family protein | -0.779751921 |
|  | CNAG_04837 | *MLN1* | Hypothetical protein | -0.314276256 |
|  | CNAG_04864 | *CIR1* | Iron-responsive gata-type transcription factor | -0.294243282 |
|  | CNAG_05023 | *CAS91* | Putative maltose o-acetyltransferase | -0.396029552 |
|  | CNAG_05062 | *PUt2* | Hypothetical protein | -0.286405486 |
|  | CNAG_05081 | *PDE1* | Phosphodiesterase | -0.17141564 |
|  | CNAG_05139 | *UGT1* | Solute carrier family 35 (UDP-sugar transporter), member A1/2/3 | 0.135099801 |
|  | CNAG_05144 | *CAN2* | Carbonic anhydrase | -0.881416777 |
|  | CNAG_05222 | *NRG1* | Transcriptional regulator Nrg1 | -0.265150815 |
|  | CNAG_05292 | *TPS1* | Alpha-alpha trehalose-phosphate synthase | 0.143968922 |
|  | CNAG_05301 | *CRN1* | Deoxyuridine 5'-triphosphate nucleotidohydrolase | 0.317411136 |
|  | CNAG_05351 | *PMM1* | Putative phosphomannomutase | -0.450252429 |
|  | CNAG_05372 | *BLP5* | Pr4/barwin domain protein | -0.285252171 |
|  | CNAG_05392 | *ZAP104* | Specific RNA polymerase II transcription factor | -0.383843744 |
|  | CNAG_05395 | *VAM6* | Rab guanyl-nucleotide exchange factor | 0.107795053 |
|  | CNAG_05420 |  | RNA polymerase II transcription factor | -0.435459325 |
|  | CNAG_05431 | *RIM101* | pH-response transcription factor PacC/Rim101 | 0.181521283 |
|  | CNAG_05465 | *GIB2* | Guanine nucleotide-binding protein subunit beta-like protein | -0.316312202 |
|  | CNAG_05538 | *JJJ1* | Hypothetical protein | -0.534034987 |
|  | CNAG_05562 | *PBX2* | Parallel beta-helix repeat protein | -0.251679337 |
|  | CNAG_05622 | *RPH1* | Specific transcriptional repressor | 0.401110321 |
|  | CNAG_05817 | *GMT1* | GDP-mannose transporter 1 | -0.2343137 |
|  | CNAG_05886 | *MMS2* | Ubiquitin-conjugating enzyme E2 | 0.086371594 |
|  | CNAG_05934 | *LIV15* | Translin domain protein | -0.23861475 |
|  | CNAG_06016 | *CAP6* | Hypothetical protein | 0.139069648 |
|  | CNAG_06031 | *KRE63* | Putative beta-glucan synthase | -0.238687104 |
|  | CNAG_06205 | *BLP3* | Pr4/barwin domain protein | -0.242391202 |
|  | CNAG_06241 | *CFO1* | Ferroxidase/laccase | 0.168354895 |
|  | CNAG_06283 | *LIV4* | Putative myb-like mRNA polymerase I termination factor | -0.479281412 |
|  | CNAG_06322 | *SAS3* | Hypothetical protein | -0.487853855 |
|  | CNAG_06346 | *BLP1* | Hypothetical protein | -1.18150677 |
|  | CNAG_06464 | *LIV7* | Hypothetical protein | 0.183652803 |
|  | CNAG_06524 | *FRE3* | Hypothetical protein | 4.348573694 |
|  | CNAG_06574 | *APP1* | Secreted antiphagocytic protein | 1.11477572 |
|  | CNAG_06591 | *SET302* | Hypothetical protein | -0.329713198 |
|  | CNAG_06655 | *GPI8* | Phosphatidylinositol glycan, class K | 0.223006074 |
|  | CNAG_06762 | *GAT204* | Hypothetical protein | -0.393815042 |
|  | CNAG_06832 | *KRE62* | Putative beta-glucan synthase | -1.227727762 |
|  | CNAG_06835 | *KRE61* | Putative beta-glucan synthase | -0.166405328 |
|  | CNAG_06980 | *STE11alpha* | MAPKK kinase, Ste/Ste11 protein kinase | 0.317591405 |
|  | CNAG_07507 | *STE50* | Protein kinase regulator | -0.274868604 |
|  | CNAG_07680 | *HAP5* | Transcriptional activator *HAP5* | 0.131411312 |
|  | CNAG_07701 | *CTR2* | Putative copper ion transporter | 0.528660759 |
| Melanin | | | | |
|  | CNAG_00130 | *RCK2* | Predicted protein kinase | 0.448163545 |
|  | CNAG_00193 | *GAT1* | Gata type zinc finger protein | -0.215402435 |
|  | CNAG_00293 | *RAS1* | Ras-like protein | -0.198864829 |
|  | CNAG_00375 | *GCN5* | Saga complex histone acetyltransferase | -0.338704771 |
|  | CNAG_00415 | *CDC2801* | Cyclin-dependent kinase of PITSLRE subfamily | -0.327495165 |
|  | CNAG_00440 | *SSN801* | Cyclin subunit of mediator subcomplex | -0.237369278 |
|  | CNAG_00654 | *SRX1* | Sulfiredoxin | -0.314693713 |
|  | CNAG_00740 | *SNF5* | Swi/Snf chromatin-remodeling complex subunit | -0.374848103 |
|  | CNAG_00757 | *DOA4* | Ubiquitin carboxyl-terminal hydrolase 8 | 0.195896704 |
|  | CNAG_00979 | *CTR4* | Solute carrier family 31 (copper transporter) | -0.326577931 |
|  | CNAG_01172 | *PBX1* | Parallel beta-helix repeat protein | -0.923196318 |
|  | CNAG_01242 | *HAPX* | Hypothetical protein | 0.092345969 |
|  | CNAG_01644 | *MLN4* | Protein of unknown function | -0.251630786 |
|  | CNAG_01678 | *NHA1* | Na+/H+ antiporter | -0.248824993 |
|  | CNAG_02048 | *PUT5* | Proline oxidase | -0.866105271 |
|  | CNAG_02215 | *HAP3* | Transcriptional activator | -0.184025245 |
|  | CNAG_02236 | *PPG1* | Type 2a-like serine/threonine-protein phosphatase | 0.105788395 |
|  | CNAG_02434 | *ATX1* | Putative copper ion transporter | -0.164690738 |
|  | CNAG_02579 |  | Hypothetical protein | 0.229889986 |
|  | CNAG_02745 | *DCR2* | Hypothetical protein | -0.166649223 |
|  | CNAG_02805 | *CAN1* | Carbonic anhydrase | 0.381708027 |
|  | CNAG_02810 | *PUF4* | Pumilio-family mRNA binding protein | -0.630085894 |
|  | CNAG_02827 | *RUB1* | Ubiquitin-like protein Nedd8 | -0.524049637 |
|  | CNAG_02867 | *PLC1* | Phosphatidylinositol phospholipase C, delta | 0.555914105 |
|  | CNAG_03188 | *SET202* | Histone-lysine N-methyltransferase, H3 lysine-36 specific | -0.252801765 |
|  | CNAG_03345 | *SSD1* | Putative translational repressor | 0.408348255 |
|  | CNAG_03409 | *SKN7* | Response regulator and transcription factor | 0.382459838 |
|  | CNAG_03464 | *LAC2* | Laccase | -0.253171475 |
|  | CNAG_03465 | *LAC1* | Laccase | -0.509427708 |
|  | CNAG_03648 | *KRE5* | UDP-glucose:glycoprotein glucosyltransferase | 0.166237931 |
|  | CNAG_03670 | *IRE1* | Serine/threonine-protein kinase, unfolded protein response | -0.182448298 |
|  | CNAG_03872 | *UBP7* | Ubiquitin carboxyl-terminal hydrolase | 0.115163906 |
|  | CNAG_03917 | *NUP75* | Nuclear pore complex protein Nup85 | -0.380589798 |
|  | CNAG_04090 | *ATF1* | Activating transcription factor | 0.615132694 |
|  | CNAG_04215 | *MET3* | Sulfate adenylyltransferase | -0.482042119 |
|  | CNAG_04453 | *IPC1* | Inositolphosphorylceramide synthase | -0.509292764 |
|  | CNAG_04552 | *ITR1A* | Putative inositol transporter | 2.989502587 |
|  | CNAG_04609 | *AGO1* | Argonaute protein | -0.18555876 |
|  | CNAG_04637 | *MBF1* | Multiprotein-bridging factor 1 | 0.249120071 |
|  | CNAG_04678 | *YPK1* | Protein kinase | 0.112244349 |
|  | CNAG_04761 | *RAS2* | Ras family protein | -0.779751921 |
|  | CNAG_04837 | *MLN1* | Hypothetical protein | -0.314276256 |
|  | CNAG_04864 | *CIR1* | Iron-responsive gata-type transcription factor | -0.294243282 |
|  | CNAG_05062 | *PUt2* | Hypothetical protein | -0.286405486 |
|  | CNAG_05081 | *PDE1* | Phosphodiesterase, phosphodiesterase, variant | -0.17141564 |
|  | CNAG_05144 | *CAN2* | Carbonic anhydrase | -0.881416777 |
|  | CNAG_05292 | *TPS1* | Alpha-alpha trehalose-phosphate synthase | 0.143968922 |
|  | CNAG_05392 | *ZAP104* | Specific RNA polymerase II transcription factor | -0.383843744 |
|  | CNAG_05395 | *VAM6* | Rab guanyl-nucleotide exchange factor | 0.107795053 |
|  | CNAG_05431 | *RIM101* | pH-response transcription factor pacC/Rim101 | 0.181521283 |
|  | CNAG_05465 | *GIB2* | Guanine nucleotide-binding protein subunit beta-like protein | -0.316312202 |
|  | CNAG_05562 | *PBX2* | Parallel beta-helix repeat protein | -0.251679337 |
|  | CNAG_06346 | *BLP1* | Hypothetical protein | -1.18150677 |
|  | CNAG_06415 | *CCC2* | Cu2 -exporting ATPase | 0.152106422 |
|  | CNAG_06524 | *FRE3* | Hypothetical protein | 4.348573694 |
|  | CNAG_06591 | *SET302* | Hypothetical protein | -0.329713198 |
|  | CNAG_06762 | *GAT204* | hypothetical protein | -0.393815042 |
|  | CNAG_06980 | *STE11alpha* | MAPKK kinase, Ste/Ste11 protein kinase | 0.317591405 |
|  | CNAG_07507 | *STE50* | Protein kinase regulator | -0.274868604 |
|  | CNAG_07636 | *CSR2* | Putative chitin synthase regulator | -0.236582666 |
|  | CNAG_07680 | *HAP5* | Transcriptional activator *HAP5* | 0.131411312 |
|  | CNAG_07701 | *CTR2* | Putative copper ion transporter | 0.528660759 |
| Mating | | | | |
|  | CNAG_00193 | *GAT1* | Gata type zinc finger protein | -0.215402435 |
|  | CNAG_00293 | *RAS1* | Ras-like protein | -0.198864829 |
|  | CNAG_00597 | *DIP5* | Amino acid transporter | -0.426993155 |
|  | CNAG_00654 | *SRX1* | Sulfiredoxin | -0.314693713 |
|  | CNAG_01262 | *GPB1* | Guanine nucleotide-binding protein subunit beta | -0.14439011 |
|  | CNAG_01445 | *APG9* | Autophagy-related protein | 0.148237229 |
|  | CNAG_01446 | *HSP12* | Hypothetical protein | 2.132362407 |
|  | CNAG_01452 | *MAT3* | Mat3 pheromone repeat protein | -0.31601691 |
|  | CNAG_01647 |  | Translocation protein *SEC66* | -0.191164873 |
|  | CNAG_01730 | *STE7* | MAP kinase kinase | -0.264935377 |
|  | CNAG_02351 | *CHI4* | Hypothetical protein | 0.275434071 |
|  | CNAG_02531 | *CPK1* | Mitogen activated protein kinase | 0.566724231 |
|  | CNAG_02598 | *CHI21* | Endochitinase | 0.498745336 |
|  | CNAG_02745 | *DCR2* | Hypothetical protein | -0.166649223 |
|  | CNAG_02756 | *CDC43* | Geranylgeranyltransferase-I beta subunit | -0.21364078 |
|  | CNAG_02883 | *RAC1* | Rho family protein | -0.428370761 |
|  | CNAG_03367 | *URK1* | Uridine kinase | -0.315920466 |
|  | CNAG_03409 | *SKN7* | Response regulator and transcription factor | 0.382459838 |
|  | CNAG_03422 | *CTS1* | Calcineurin temperature suppressor Cts1 | 0.149843047 |
|  | CNAG_03938 | *CPR2* | Oheromone a factor receptor | 0.549162139 |
|  | CNAG_04119 | *ROM2* | Rho guanyl-nucleotide exchange factor | 0.13250298 |
|  | CNAG_04245 | *CHI22* | Endochitinase | -0.231053268 |
|  | CNAG_04521 |  | Oxidoreductase | -0.985435483 |
|  | CNAG_04609 | *AGO1* | Argonaute protein | -0.18555876 |
|  | CNAG_04730 | *GPR4* | Hypothetical protein | -0.289720147 |
|  | CNAG_04761 | *RAS2* | Ras family protein | -0.779751921 |
|  | CNAG_04864 | *CIR1* | Iron-responsive gata-type transcription factor | -0.294243282 |
|  | CNAG_04869 | *PNB1* | Para-nitrobenzyl esterase | -1.019295336 |
|  | CNAG_05222 | *NRG1* | Transcriptional regulator Nrg1 | -0.265150815 |
|  | CNAG_05465 | *GIB2* | Guanine nucleotide-binding protein subunit beta-like protein | -0.316312202 |
|  | CNAG_05558 | *KIN4* | Serine/threonine protein kinase | -0.215644655 |
|  | CNAG_05866 | *PRM1* | Putative plasma membrane fusion protein | 1.12944946 |
|  | CNAG_05925 | *CDC3* | Septin ring protein | -0.208014522 |
|  | CNAG_05970 | *PAK1* | Ste/Ste20/PakA protein kinase | 0.070285615 |
|  | CNAG_06765 | *LMP1* | Low mating performance protein | -0.519529745 |
|  | CNAG_06806 | *ETF1alpha* | Electron transfer flavoprotein alpha subunit | -0.523135543 |
|  | CNAG_06808 | *STE3alpha* | Pheromone a factor receptor | -0.657125254 |
|  | CNAG_06811 | *RPL22alpha* | Large subunit ribosomal protein L22e | -0.406283818 |
|  | CNAG_06812 | *SPO14alpha* | Phospholipase D1 | -0.323123919 |
|  | CNAG_06813 | *CAP1alpha* | Hypothetical protein | -0.701589794 |
|  | CNAG_06980 | *STE11alpha* | MAPKK kinase, Ste/Ste11 protein kinase | 0.317591405 |
|  | CNAG_07407 | *MFalpha3* | Mating-type pheromone alpha | -0.974668369 |
|  | CNAG_07409 | *RPO41alpha* | DNA-directed mRNA polymerase, mitochondrial | -0.66659612 |
|  | CNAG_07410 | *CID1alpha* | Hypothetical protein | -0.197735218 |
|  | CNAG_07413 | *NOG2* | Putative nucleolar GTP-binding protein | -0.412810506 |
|  | CNAG_07507 | *STE50* | Protein kinase regulator | -0.274868604 |
| Protease | | | | |
|  | CNAG_00046 |  | 26S proteasome regulatory subunit N8 | 0.280245912 |
|  | CNAG_00147 |  | Pre-mRNA-processing-splicing factor 8 | 0.260537496 |
|  | CNAG_00180 |  | Ubiquitin carboxyl-terminal hydrolase L3 | 0.472665779 |
|  | CNAG_00187 | *UBP16* | Ubiquitin carboxyl-terminal hydrolase 1 | 0.217686372 |
|  | CNAG_00455 |  | Ubiquitin carboxyl-terminal hydrolase Ubp16 | -0.468490799 |
|  | CNAG_00581 |  | Saccharopepsin | 0.406541427 |
|  | CNAG_00757 | *DOA4* | Ubiquitin carboxyl-terminal hydrolase 8 | 0.195896704 |
|  | CNAG_00989 | *UCH2* | Ubiquitin carboxyl-terminal hydrolase L5 | -0.283339391 |
|  | CNAG_01343 |  | ATP-dependent Clp protease ATP-binding subunit ClpX | 0.397082268 |
|  | CNAG_01688 |  | ATP-dependent metalloprotease | -0.454088763 |
|  | CNAG_01795 |  | Hypothetical protein | 0.356398599 |
|  | CNAG_02004 |  | OTU domain-containing protein 6B | 0.180535376 |
|  | CNAG_02239 |  | 26S protease regulatory subunit 4 | 0.447325323 |
|  | CNAG_02240 |  | Cytoplasmic protein | 0.240280635 |
|  | CNAG_02282 |  | Carboxypeptidase A4 | 0.450060236 |
|  | CNAG_03037 |  | Hypothetical protein | -0.820609904 |
|  | CNAG_03872 | *UBP7* | Ubiquitin carboxyl-terminal hydrolase | 0.115163906 |
|  | CNAG_03904 |  | 26S protease regulatory subunit 6B | 0.150163286 |
|  | CNAG_04380 |  | Peptidase | -0.391189814 |
|  | CNAG_04493 |  | Ubiquitin carboxyl-terminal hydrolase 48 | -0.604397355 |
|  | CNAG_04625 |  | Cerevisin | 0.148936278 |
|  | CNAG_04635 |  | Endopeptidase | 0.225975854 |
|  | CNAG_04666 |  | 26S protease regulatory subunit 8 | 0.186381652 |
|  | CNAG_04906 |  | 26S protease regulatory subunit 10B | 0.282517098 |
|  | CNAG_05305 |  | Hypothetical protein | 1.435589647 |
|  | CNAG_05713 |  | Elongator complex protein 1 | -0.226897296 |
|  | CNAG_05742 | *STP1* | Putative site-2 protease | -0.354935589 |
|  | CNAG_05872 |  | Endopeptidase | 0.437070994 |
|  | CNAG_06153 |  | 26S protease regulatory subunit 6A-B | 0.401220915 |
|  | CNAG_06410 |  | ATP-dependent Clp endopeptidase, proteolytic subunit ClpP | -0.20806081 |
|  | CNAG_06637 |  | Ubiquitin carboxyl-terminal hydrolase 22/27/51 | -0.230488663 |
|  | CNAG_06680 |  | *COP9* signalosome complex subunit 6 | 0.288214239 |
|  | CNAG_06983 |  | Hypothetical protein | 0.314545254 |
|  | CNAG_07520 |  | Endopeptidase | 0.661884424 |

**Supplementary Table 2.** *C. neoformans* genes analyzed in this study**.**

| Gene | FungiDB |
| --- | --- |
| *HDA1* | CNAG_01563 |
| *HOS1* | CNAG_05096 |
| *CLR61* | CNAG_01699 |
| *CLR62* | CNAG_05276 |
| *HOS3* | CNAG_00660 |
| *HOS2* | CNAG_05563 |
| *RPD3* | CNAG_05690 |

**Supplementary Table 3.** Strain list.

| Strain | Gene | Genotype | Source |
| --- | --- | --- | --- |
| H99 | - | *MAT****α*** | ^4^ |
| KN99 | - | *MAT****a*** *– MAT****α*** | ^5^ |
| H99 | *RAS1* | *ras1Δ::NEO MAT****α*** | ^6^ |
| FS3 | *HDA1* | *hda1Δ::NEO MAT****α*** | This study |
| FS5 | *HDA1* | *hda1Δ::NEO MAT****a*** | This study |
| FS4 | *hda1Δ +HDA1* | *hda1Δ::NEO +HDA1 + pCH233 (NAT) MAT****α*** | This study |
| FS8 | *HOS3* | *hos3Δ::NEO MAT****a*** | This study |
| FS11 | *HOS3* | *hos3Δ::NEO MAT****α*** | This study |
| FS9 | *HOS1* | *hos1Δ::NEO MAT****a*** | This study |
| FS10 | *HOS2* | *hos2Δ::NAT MAT****α*** | This study |
| FS12 | *CLR62* | *clr62Δ::NEO MAT****α*** | This study |
| FS13 | *RPD3* | *rpd3Δ::NAT MAT****α*** | This study |
| FS14 | *HDA1/HOS2* | *hda1Δ::NEO + hos2Δ::NAT MAT****α*** | This study |
| FS15 | *HDA1/RPD3* | *hda1Δ::NEO + rpd3Δ::NAT MAT****α*** | This study |

| \| Supplementary Table 4. Genes and primers \| \| \| \| \| \| --- \| --- \| --- \| --- \| --- \| \| Gene \| **Primer** \| **Primer**  **Code** \| **Sequence 5’- 3’** \| **Target** \| | | | | |
| --- | --- | --- | --- | --- | --- | --- | --- | --- | --- | --- | --- | --- | --- | --- |
| *NEO* | NSL | AA3934 | TCGATGCGATGTTTCGCT | NEO |
|  | NSR | AA3935 | CCTGAATGAACTGCAGGA | NEO |
| *HDA1* | P1 | AA4379 | CAAAGGCAACAGATCGGAAT | Flank 5’ |
|  | P2 | AA4380 | CCAAGCTTGGCGTAATCATGCTTGCTGTGGGTTGAGTCG | Flank 5’ + 5’ NEO |
|  | P3 | AA4385 | CGACTCAACCCACAGCAAGCATGATTACGCCAAGCTTGG | Reverse complement of P2 |
|  | P4 | AA4386 | CCCCAGCCAGCTCGCTACATGAATTGTAATACGACTCAC | Reverse complement of P5 |
|  | P5 | AA4387 | GTGAGTCGTATTACAATTCATGTAGCGAGCTGGCTGGGG | Flank 3’ + 3’ NEO |
|  | P6 | AA4388 | TCTTGTTACAGCCAGCACAACA | Flank 3’ |
|  | P9 | AA4396 | CCGTTCCTCCTTCTTCTCCAACT | ORF inside |
|  | P10 | AA4397 | TTTTCATCCTCCAATACCAC | ORF inside |
|  | P11 | AA4419 | AAGGAGATCTGACACTTACGCTCTTT | Flank 5’ + *Bgl2* |
|  | P12 | AA4420 | GTAGTCTAGAAGTTGTGTTCATCAGTCA | Flank 3’ + *Bgl2* |
|  | RT-PCR | - | CTCCTGAAGTCACCGAGCT | CNAG_01563 Forward |
|  | RT-PCR | - | GGC CAA TGG TAT TTG GTA CA | CNAG_01563 Reverse |
| *HOS1* | P1 | AA4516 | CCATGACATTAGGAGTAAGGAT | Flank 5’ |
|  | P2 | AA4517 | CCAAGCTTGGCGTAATCATGTCGGTTGTCTGTTGAGTGTCATAC | Flank 5’ + 5’ NEO |
|  | P3 | AA4518 | GTATGACACTCAACAGACAACCGACATGATTACGCCAAGCTTGG | Reverse complement of P2 |
|  | P4 | AA4519 | AAACCATCAGGAAACCATGTGAATTGTAATACGACTCAC | Reverse complement of P5 |
|  | P5 | AA4520 | GTGAGTCGTATTACAATTCACATGGTTTCCTGATGGTTT | Flank 3’ + 3’ NEO |
|  | P6 | AA4521 | TATCGTGAGTTGTAAAATGGGTG | Flank 3’ |
|  | P9 | AA4522 | CTACAGTATATCCCTATCCCTTGA | ORF inside |
|  | P10 | AA4523 | GATAAGAAGAGGGTAGAAGAGGTT | ORF inside |
|  | RT-PCR | - | ACA TAG AGC GCC TTC TAA AG | CNAG_05096 Forward |
|  | RT-PCR | - | GAT TGT CGT GCG AGA GAT T | CNAG_05096 Reverse |
| *CLR61* | P9 | AA4491 | GAATATCTTCTCGTAGTCCAGTG | ORF inside |
|  | P10 | AA4492 | TTACTGTTGCTCCTCCTACTATC | ORF inside |
|  | RT-PCR | - | TCAGACCTCGTCGAGCAACT | CNAG_01699 Forward |
|  | RT-PCR | - | CCTCTTGTCCCATTTCCAGT | CNAG_01699 Reverse |
| *CLR62* | P1 | AA4554 | TTTGCATCTCTTCTCCAGGA | Flank 5’ |
|  | P2 | AA4555 | CCAAGCTTGGCGTAATCATGCGGACGTAACAAGGAGTTGAGA | Flank 5’ + 5’ NEO |
|  | P3 | AA4556 | TCTCAACTCCTTGTTACGTCCGCATGATTACGCCAAGCTTGG | Reverse complement of P2 |
|  | P4 | AA4557 | GAGACGTGATAGATAATAGGAGGAGAATTGTAATACGACTCAC | Reverse complement of P5 |
|  | P5 | AA4558 | GTGAGTCGTATTACAATTCTCCTCCTATTATCTATCACGTCTC | Flank 3’ + 3’ NEO |
|  | P6 | AA4559 | TTATCCTGCAAAGCGTCTAACCT | Flank 3’ |
|  | P9 | AA4568 | GTGATGTCAGAGTGAGTTCTTTT | ORF inside |
|  | P10 | AA4569 | TTACTTGATACGCTCCAGATACT | ORF inside |
|  | RT-PCR | - | TTA AAC CTC GAA GGG CTA CC | CNAG_05276 Forward |
|  | RT-PCR | - | TAA AGC ATC CGC AGT CTC C | CNAG_05276 Reverse |
| *HOS3* | P1 | AA4479 | TTGCCTGGATTGGTCTTTTC | Flank 5’ |
|  | P2 | AA4480 | CCAAGCTTGGCGTAATCATGTCTCCCCCTTTTCCTCTTTC | Flank 5’ + 5’ NEO |
|  | P3 | AA4481 | GAAAGAGGAAAAGGGGGAGACATGATTACGCCAAGCTTGG | Reverse complement of P2 |
|  | P4 | AA4483 | TATCCTCCTCAATTCCATAGTCGAATTGTAATACGACTCAC | Reverse complement of P5 |
|  | P5 | AA4482 | GTGAGTCGTATTACAATTCGACTATGGAATTGAGGAGGATA | Flank 3’ + 3’ NEO |
|  | P6 | AA4484 | GTTTGTCAGAAAGTCAGAAAGC | Flank 3’ |
|  | P9 | AA4489 | CTTATGCGGATGATATACAGGT | ORF inside |
|  | P10 | 444490 | GAGTGCAAGATATAGAGGGTAGAT | ORF inside |
|  | RT-PCR | - | TGGGCAGTACATCGAAAACATT | CNAG_00660 Forward |
|  | RT-PCR | - | GGCTTCAGTCTGTTCCATGA | CNAG_00660 Reverse |
| *HOS2* | P1 | AA4560 | CTTCCCAATGTGAGACTTAAATA | Flank 5’ |
|  | P6 | AA4561 | TAAACTGGAGTTATCATCGTACC | Flank 3’ |
|  | P9 | AA4574 | CTAACAAAAGACTGGACGAAAT | ORF inside |
|  | P10 | AA4575 | AATAGTTGTTTTGTGGGAGTTC | ORF inside |
|  | RT-PCR | - | ATG GGG AAC GCC ATC CTA T | CNAG_05563 Forward |
|  | RT-PCR | - | AGG GGC ATG ATA CGA CAT TT | CNAG_05563 Reverse |
| *RPD3* | P1 | AA4562 | CGTCTAAATAGGATATGGATGAC | Flank 5’ |
|  | P6 | AA4563 | CTATGCGTCTATTACCGTAAGTT | Flank 3’ |
|  | P9 | AA4572 | TTTTGTACGTGTTCAGCATATT | ORF inside |
|  | P10 | AA4573 | CGATTCCGTTATCTCTTACTTC | ORF inside |
|  | RT-PCR | - | GCAAAGGTAAAGGATACGCT | CNAG_05690 Forward |
|  | RT-PCR | - | CACCTGGTTGATACCATTCG | CNAG_05690 Reverse |
